# Supplementary material for: Whole-exome sequencing study identifies rare variants and genes associated with intraocular pressure and glaucoma
Source: Nat Commun. 2022 Nov 30;13:7376. doi: 10.1038/s41467-022-35188-3 (PMC9712679; doi:10.1038/s41467-022-35188-3)
Supplement: Supplementary file 1 — Supplementary Information [file 41467_2022_35188_MOESM1_ESM.docx]

**Supplementary information**

**Whole-exome sequencing study identifies rare variants and genes associated with intraocular pressure and glaucoma**

Xiaoyi Raymond Gao^1,2,3,4*^, Marion Chiariglione^1^, Alexander J. Arch^1^

^1^Department of Ophthalmology and Visual Sciences, The Ohio State University, Columbus, OH 43210, USA

^2^Department of Biomedical Informatics, The Ohio State University, Columbus, OH 43210, USA

^3^Division of Human Genetics, The Ohio State University, Columbus, OH 43210, USA

^4^Ohio State University Physicians Inc., Columbus, OH, USA

^*^Correspondence should be addressed to: [raymond.gao@osumc.edu](mailto:raymond.gao@osumc.edu)

**Supplementary Table 1. Rare variants with *P* < 5 x 10^-7^ from pan-ancestry single-variant analysis used in rvPRS.**

| **rsID** | **Effect Allele** | **Gene** | **Function** | **Weight** |
| --- | --- | --- | --- | --- |
| 1:11193627:G:T | G | *ANGPTL7* | nonsynonymous | 1 |
| 1:156382152:C:G | G | *RHBG* | nonsynonymous | 1 |
| 1:171636338:G:A | A | *MYOC* | stopgain | 5 |
| 2:6887057:T:C | C | *RSAD2* | nonsynonymous | 1 |
| 2:86482074:G:A | A | *KDM3A* | nonsynonymous | 1 |
| 3:160439089:T:C | C | *TRIM59* | nonsynonymous | 1 |
| 4:13613559:T:C | C | *BOD1L1* | nonsynonymous | 1 |
| 5:73448094:G:T | T | *FOXD1* | nonsynonymous | 1 |
| 5:75147406:C:T | T | *ANKRD31* | nonsynonymous | 1 |
| 5:140882996:A:G | G | *PCDHA13* | nonsynonymous | 1 |
| 5:179032549:C:T | T | *ZNF879* | nonsynonymous | 1 |
| 8:84887679:C:T | T | *RALYL* | nonsynonymous | 1 |
| 9:137010979:C:T | T | *ABCA2* | nonsynonymous | 1 |
| 10:73911900:C:A | A | *PLAU* | nonsynonymous | 1 |
| 11:824755:G:A | A | *PNPLA2* | nonsynonymous | 1 |
| 12:111692710:A:G | G | *ACAD10* | startloss | 2 |
| 14:50188639:G:C | G | *SOS2* | nonsynonymous | 1 |
| 14:103126575:C:G | G | *TNFAIP2* | nonsynonymous | 1 |
| 16:48086800:C:T | T | *ABCC12* | nonsynonymous | 1 |
| 18:63637790:G:C | C | *SERPINB4* | nonsynonymous | 1 |
| 19:1529427:G:T | T | *PLK5* | nonsynonymous | 1 |
| 19:24126560:G:T | T | *ZNF254* | nonsynonymous | 1 |
| 19:48311573:C:T | T | *CCDC114* | nonsynonymous | 1 |
| 21:32453387:G:A | A | *EVA1C* | nonsynonymous | 1 |
| 22:50277616:G:A | A | *PLXNB2* | nonsynonymous | 1 |

REGENIE was used to perform single-variant association tests (two-sided). We extracted rare variants with *P* < 5 x 10^-7^. No adjustments were made for multiple comparisons.

**Supplementary Table 2. FinnGen glaucoma phenotypes and their extraction criteria.**

| **FinnGen glaucoma phenotypes** | **Endpoint definitions** |
| --- | --- |
| Glaucoma | H40\|H42 (ICD-10) |
| Use of antiglaucoma preparations and miotics | Medicine purchases: ATC S01EA, Min. number of events: 3 |
| Primary open-angle glaucoma | H40.1 (ICD-10), 3651 (ICD-9), 3751[0-3] (ICD-8) |

Source of endpoint definitions: https://risteys.finngen.fi/

**Supplementary Table 3. Sub-population single-variant analysis results.**

| **Sub-population** | **Chr** | **Pos** | **rsID** | **A0** | **A1** | **A1FREQ** | **N** | **Beta** | **SE** | **CHISQ** | ***P*** | **Gene** |
| --- | --- | --- | --- | --- | --- | --- | --- | --- | --- | --- | --- | --- |
| Asian | 1 | 1708342 | rs556417493 | C | G | 0.00079957 | 3752 | 6.51 | 1.13 | 33.38 | 7.59E-09 | *CDK11A* |
|  |  |  |  |  |  |  |  |  |  |  |  |  |
| Black | 19 | 1529427 | rs776910868 | G | T | 0.00106512 | 3286 | 8.15 | 1.33 | 37.66 | 8.42E-10 | *PLK5* |
|  |  |  |  |  |  |  |  |  |  |  |  |  |
| Non-white | 1 | 244432616 | rs375507039 | T | C | 0.00025956 | 11558 | 7.65 | 1.33 | 32.88 | 9.82E-09 | *ADSS2* |
|  | 10 | 73911900 | rs367716060 | C | A | 0.00021578 | 11586 | 8.25 | 1.46 | 31.91 | 1.62E-08 | *PLAU* |
|  | 14 | 72670683 | rs933632776 | AC | A | 0.00021582 | 11584 | 8.93 | 1.46 | 37.40 | 9.62E-10 | *DPF3* |
|  |  |  |  |  |  |  |  |  |  |  |  |  |
| Non-white | 6 | 31356961 | rs201956837 | G | A | 0.00030651 | 11419 | 4.47 | 1.24 | 13.06 | 0.00030241 | *HLA-B* |
| white | 6 | 31356961 | rs201956837 | G | A | 5.61E-05 | 97987 | 4.33 | 0.97 | 20.00 | 7.76E-06 | *HLA-B* |

REGENIE was used to perform single-variant association tests (two-sided). No adjustments were made for multiple comparisons. Abbreviations: Chr, chromosome; Pos, position; A0, allele 0; A1, allele 1; A1Freq, allele 1 frequency in the analyzed sample; SE, standard error; CHISQ, chi-square.

**Supplementary Figure 1. Quantile-quantile plots for single-variant results.**

Quantile-quantile plots shows adequate control of population stratification. (a) White-only single-variant association results. (b) Pan-ancestry single-variant association results.

(a)

**
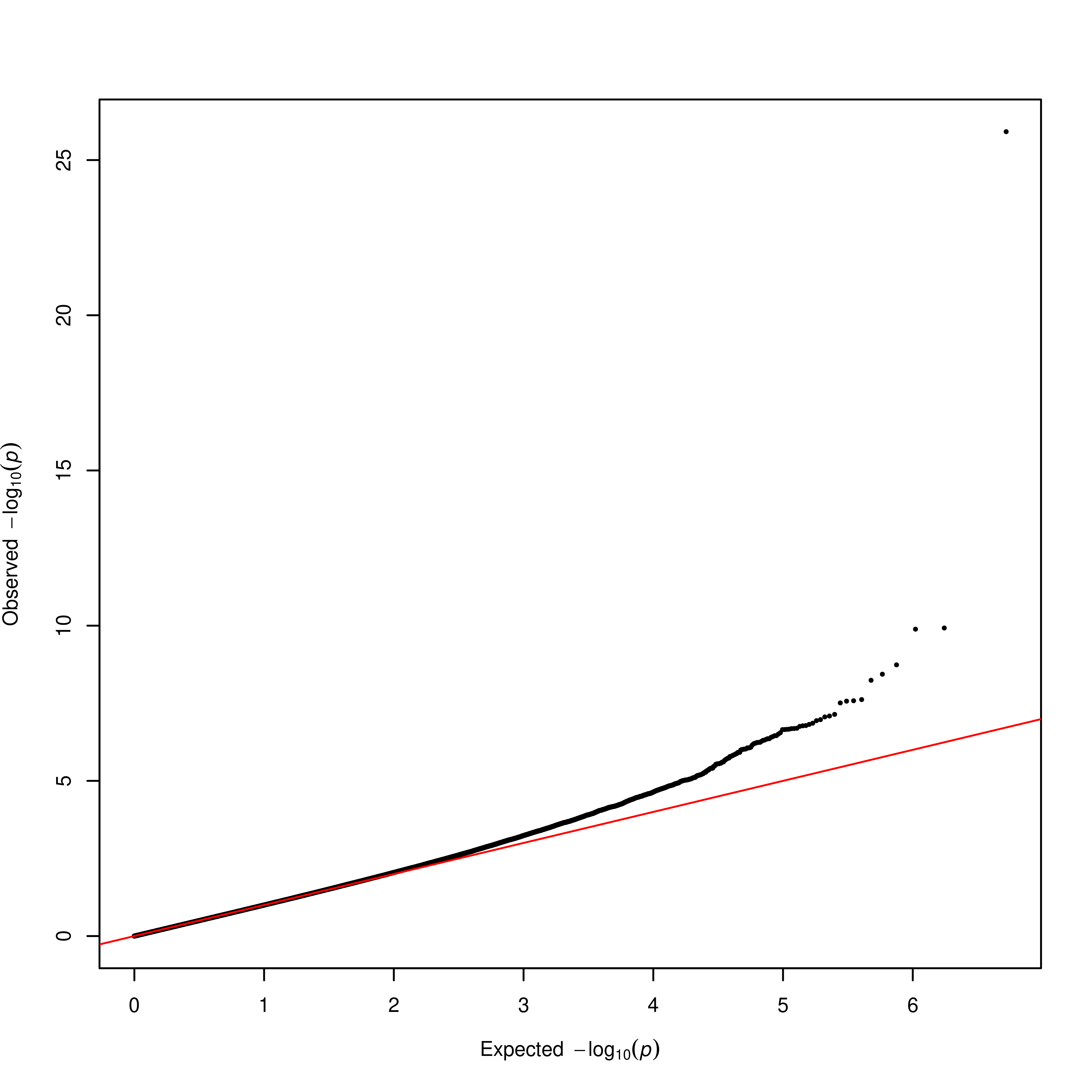
**

(b)

**
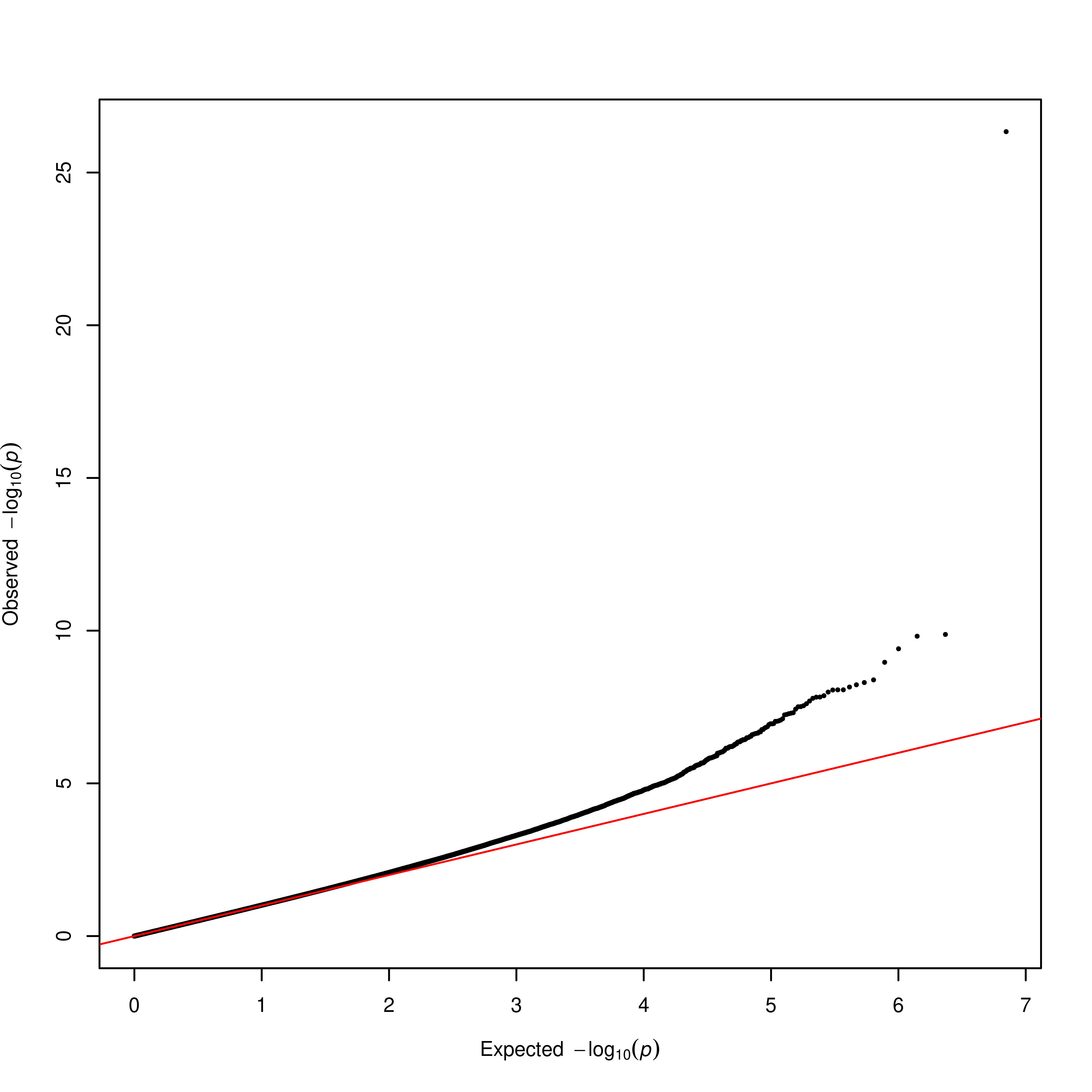
**

**Supplementary Figure 2. Gene expression profiles from Genevestigator.**

Gene expression profiles were generated with GENEVESTIGATOR^®^ (source: Hruz T*, et al.* Genevestigator v3: a reference expression database for the meta-analysis of transcriptomes. *Adv Bioinformatics* **2008**, 420747 (2008). https://doi.org/10.1155/2008/420747). Genes are listed alphabetically according to gene name.

*AAK1*

*
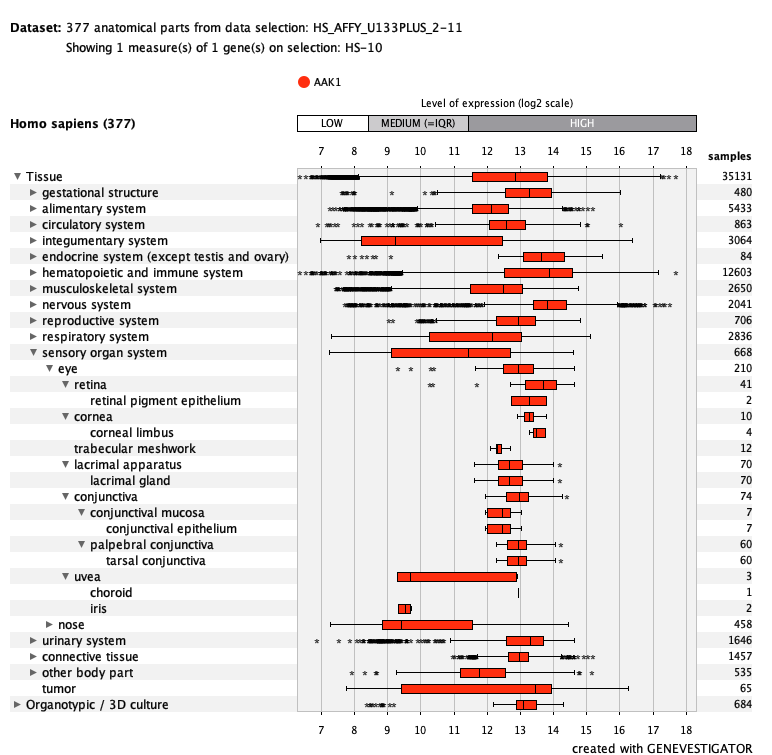
*

*ABI3BP*

*
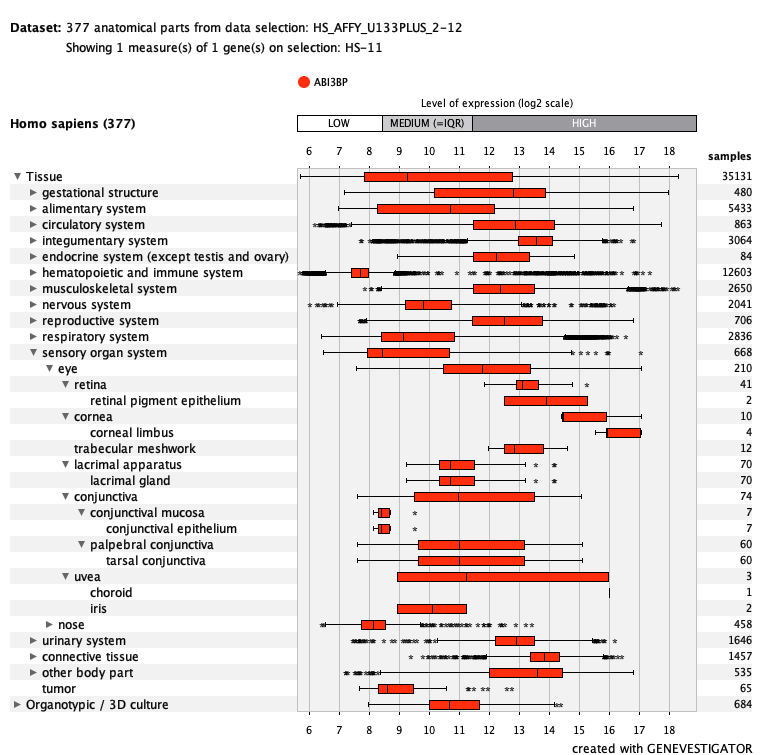
*

*ACAD10*

*
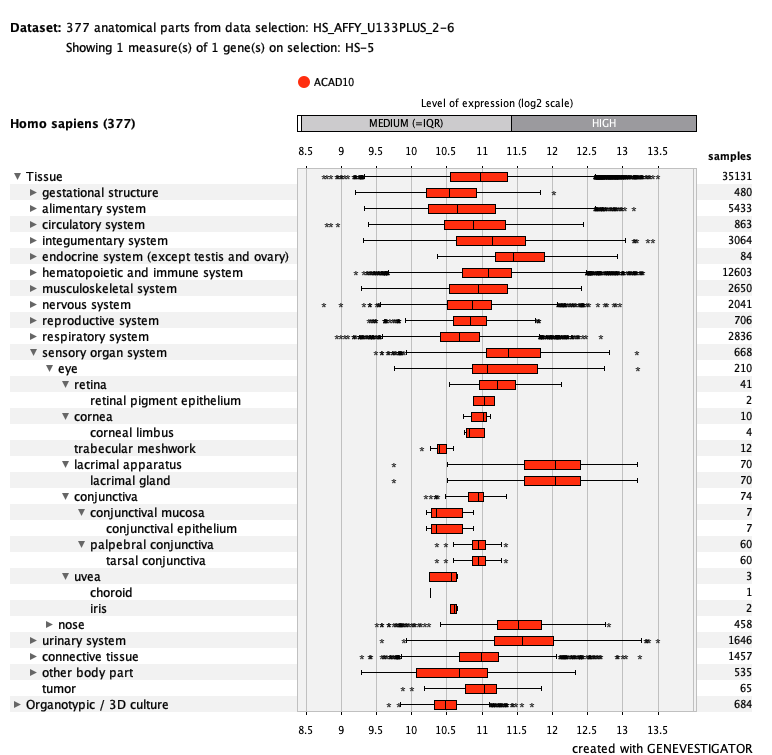
*

*ADRB1*

*
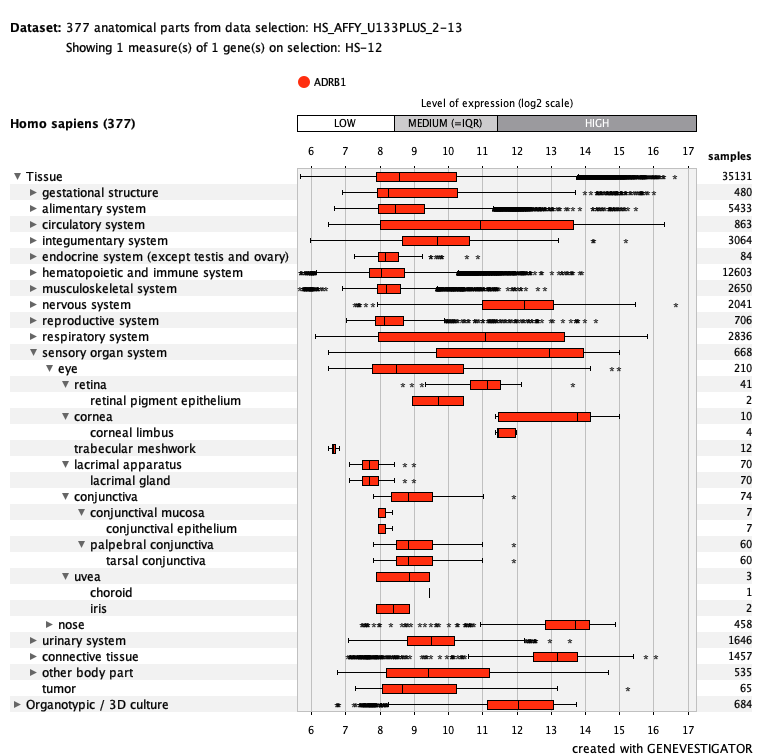
*

**
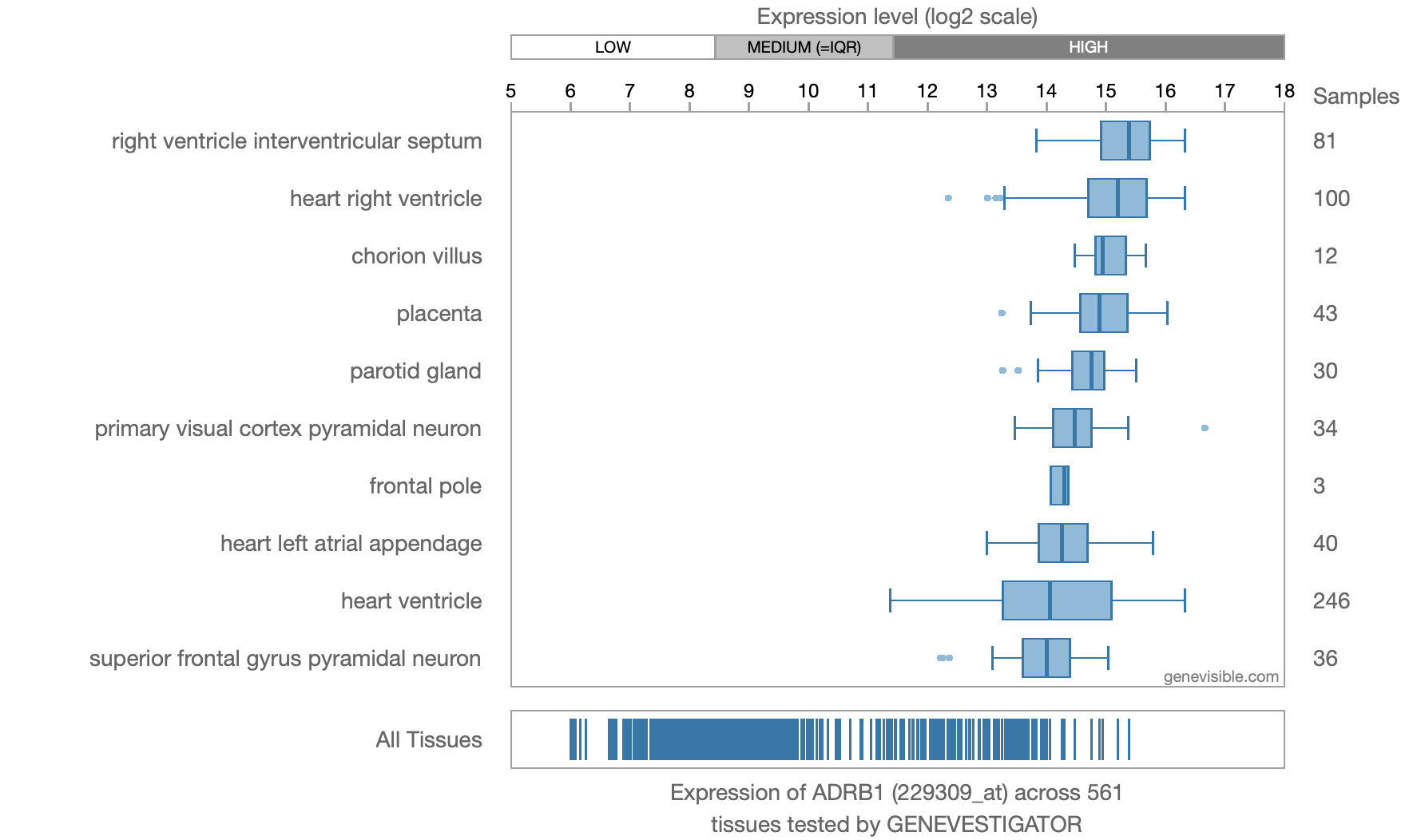
**

*ADSS*

*
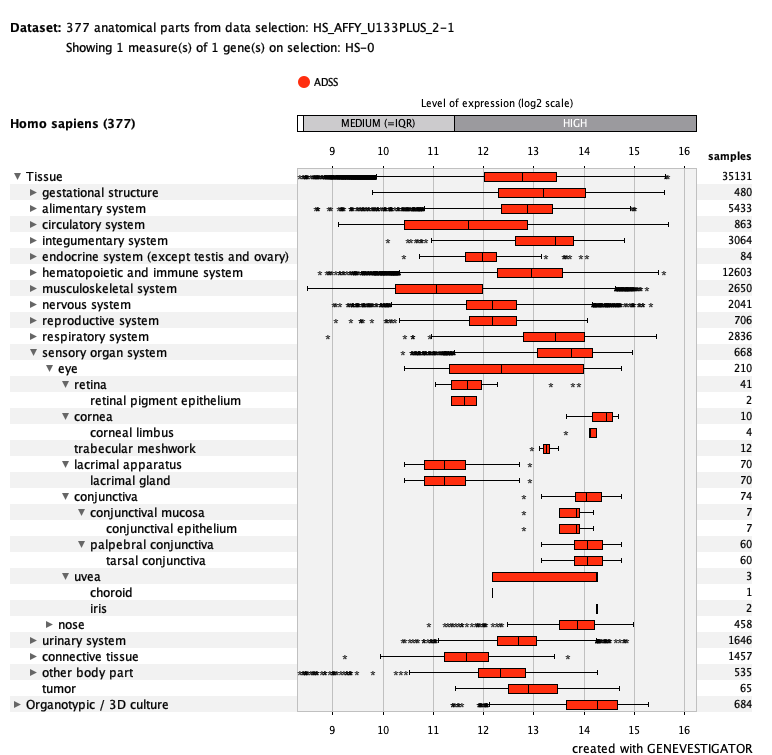
*

*ANGPTL7*

*
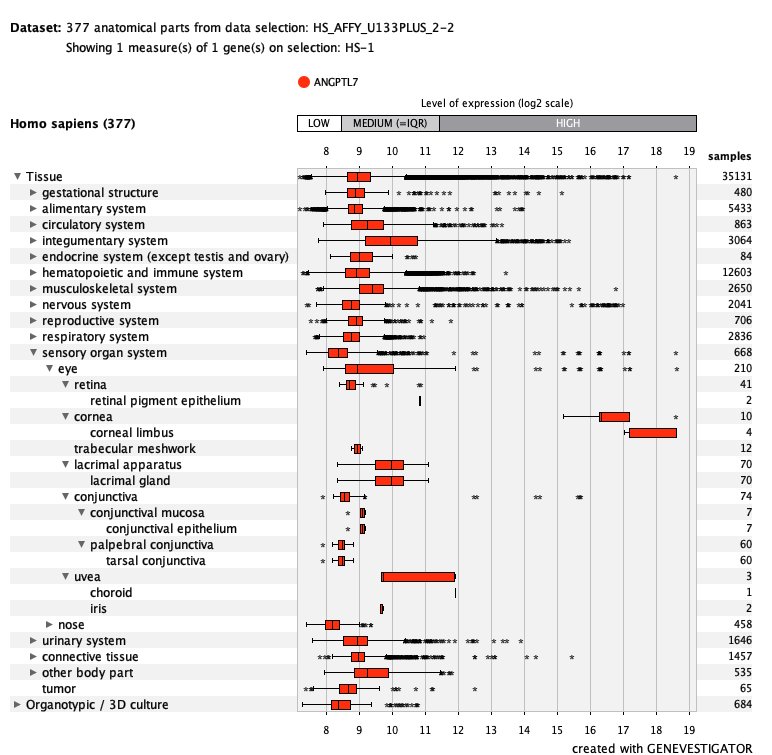
*

*BOD1L1*

*
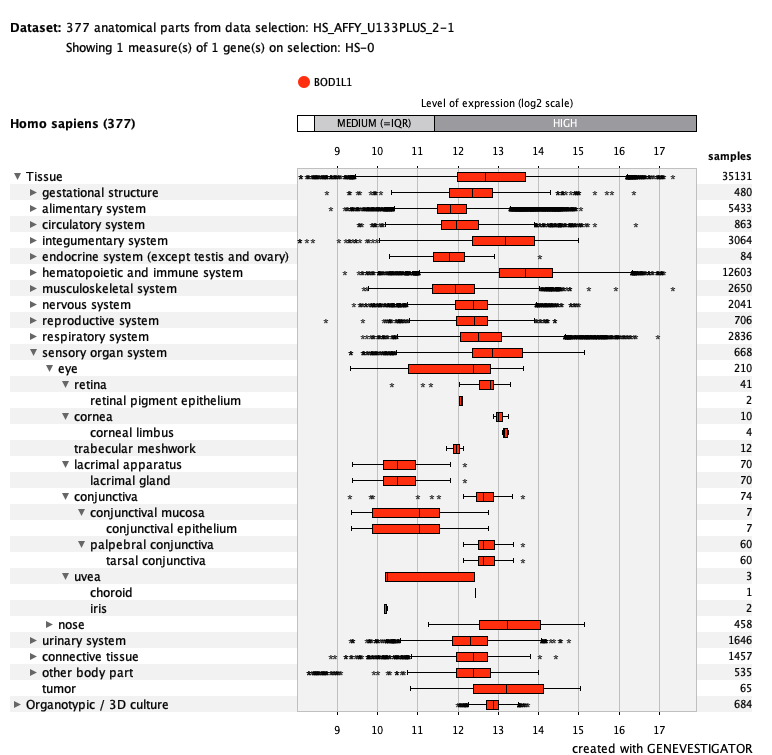
*

*CDCA8*

*
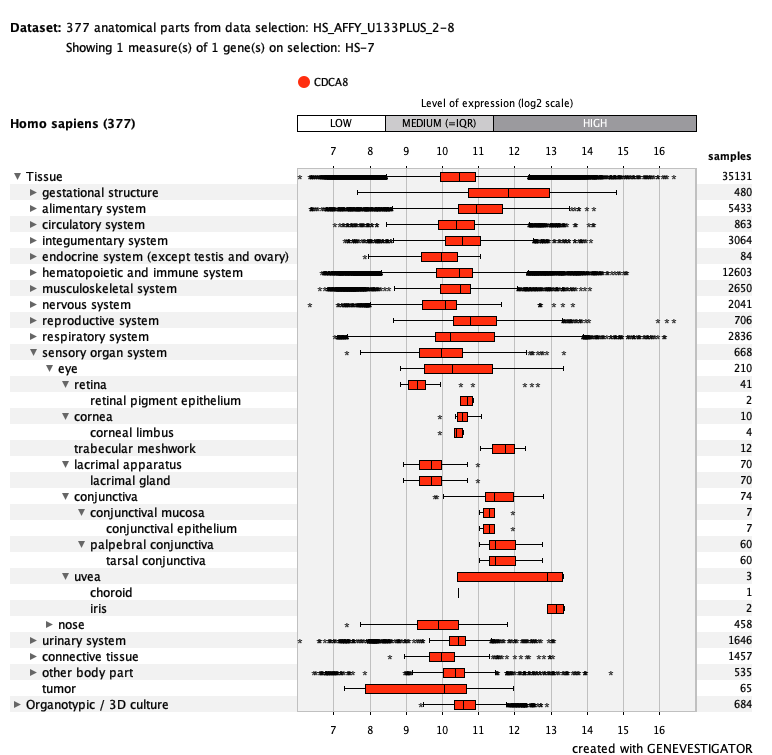
*

*CDK11A*


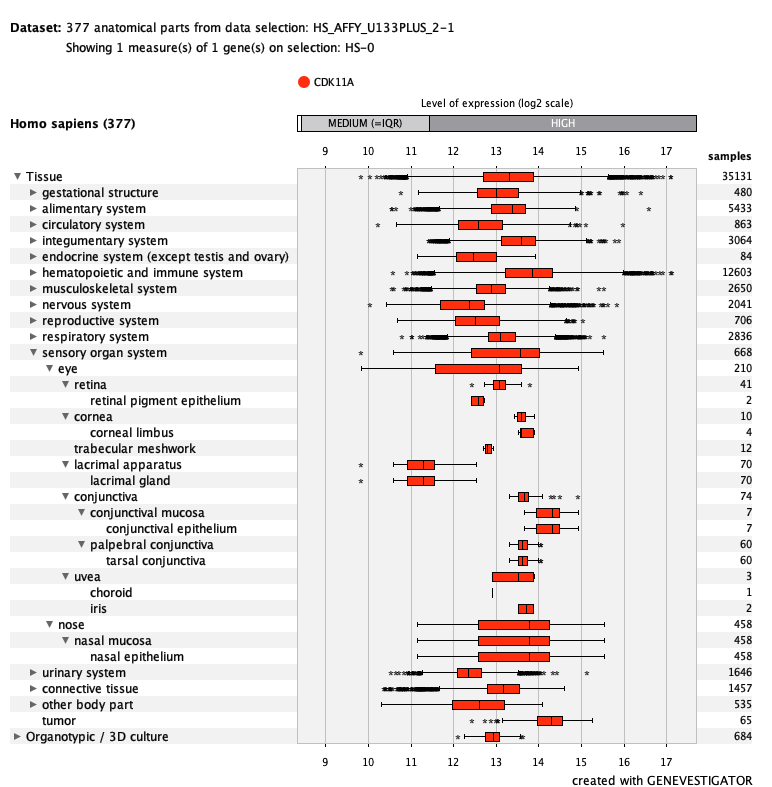


*CFAP298*

*
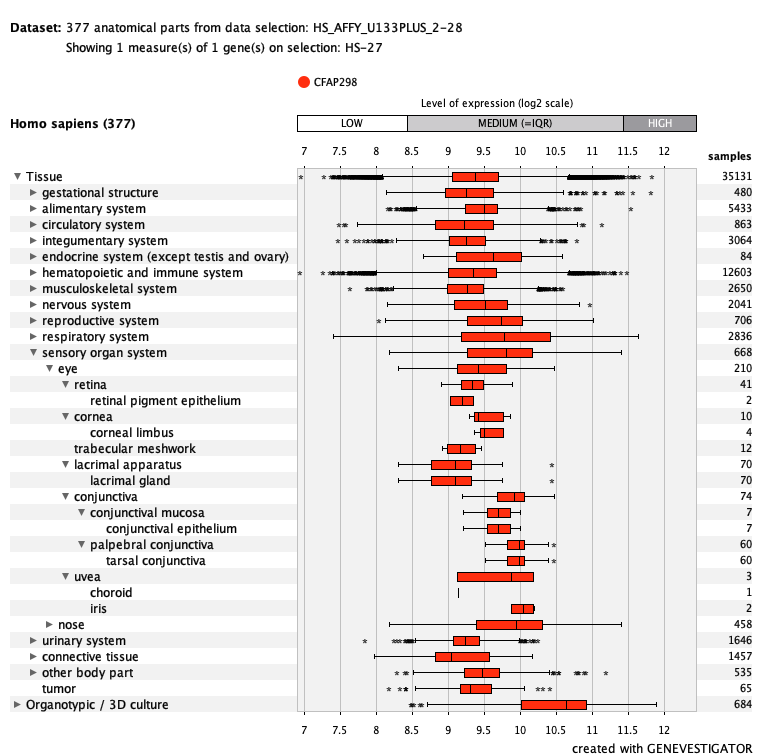
*

*DACT3*

*
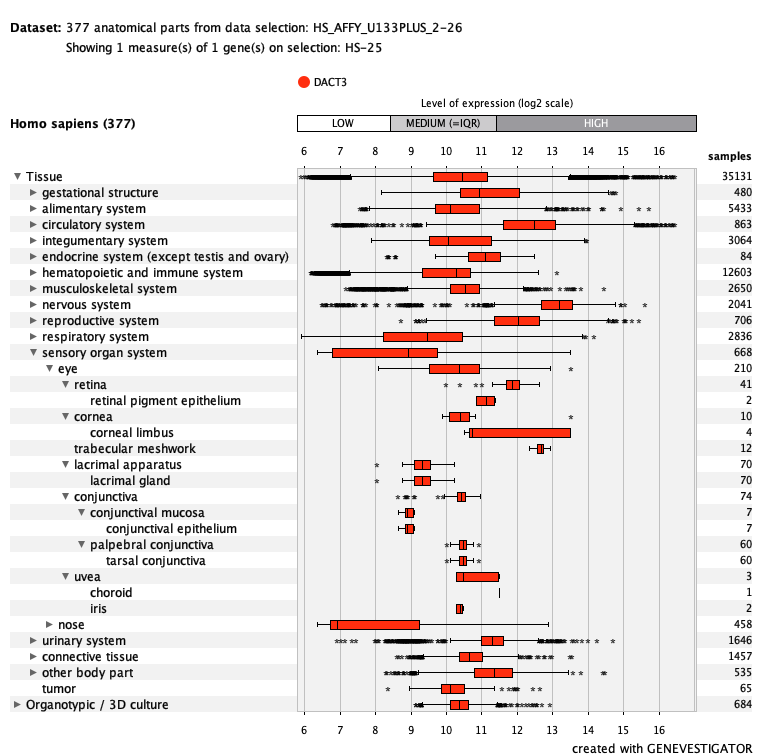
*

*DNTT*

*
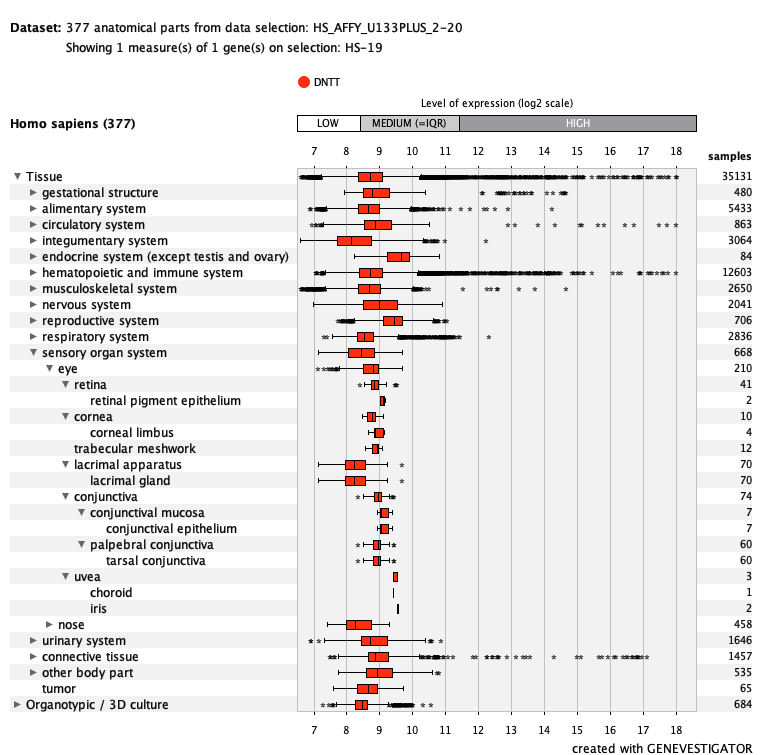
*

*DOK4*

*
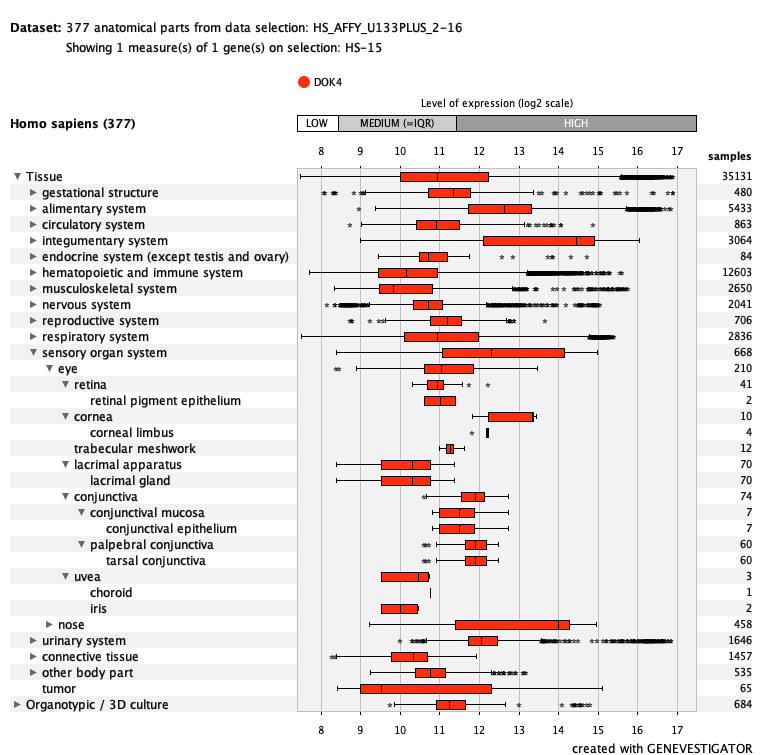
*

*DPF3*

*
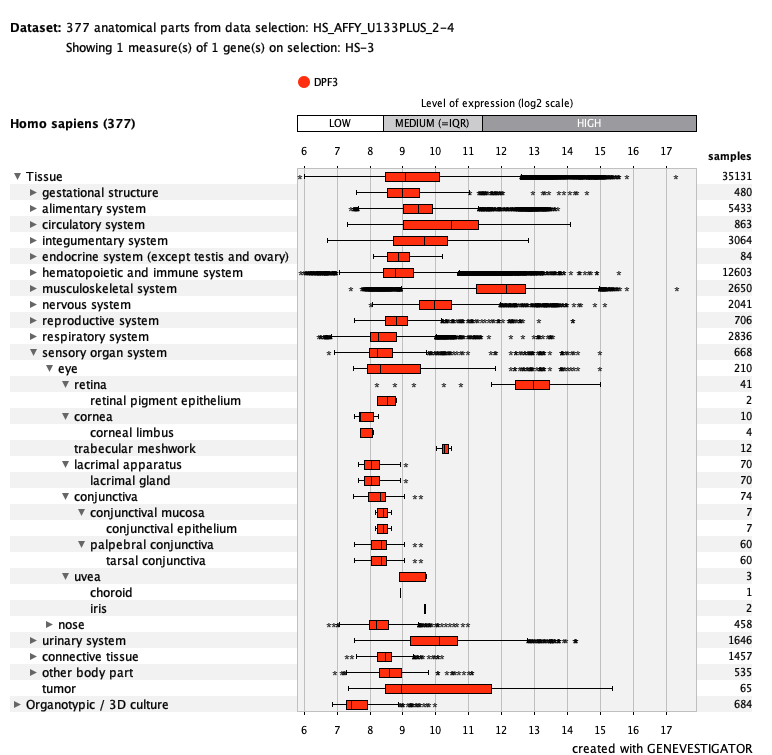
*

*EHMT1*

*
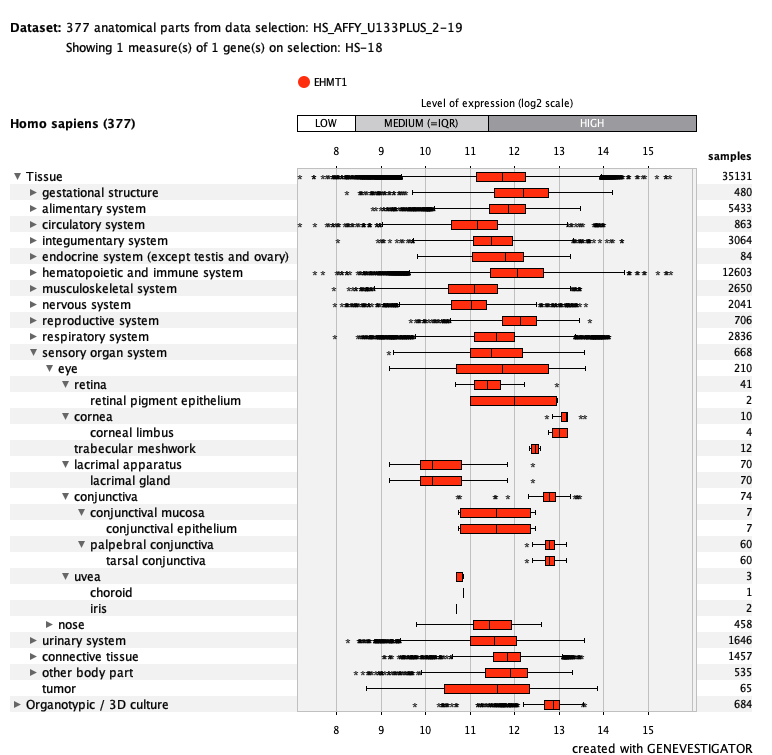
*

*EVA1C*

*
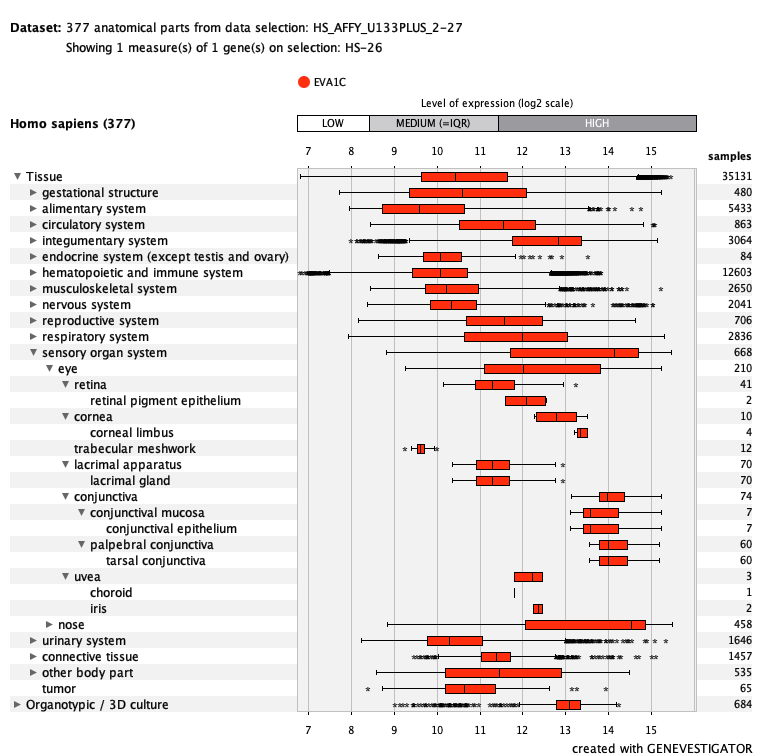
*

*FOXD1*

*
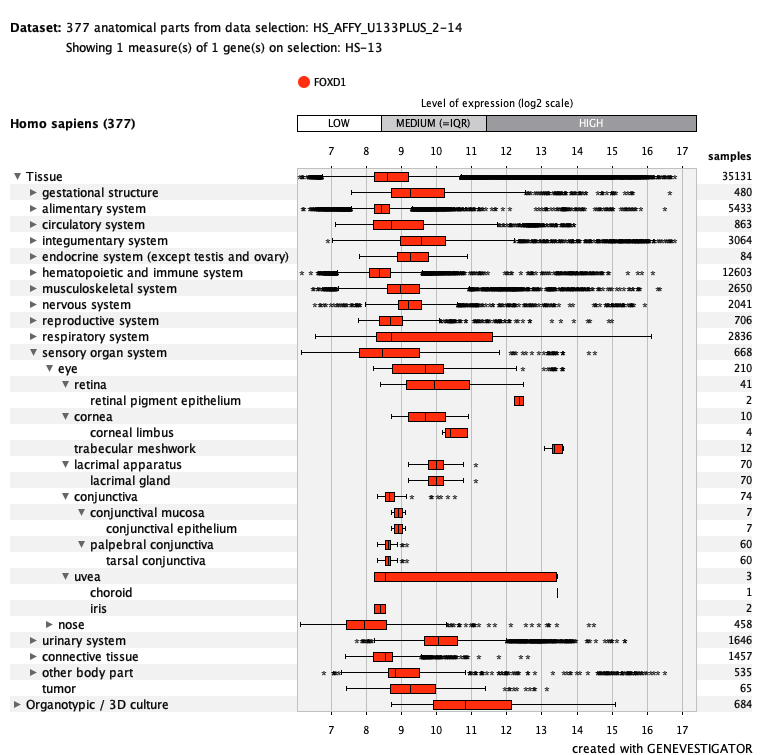
*

*GUSB*

*
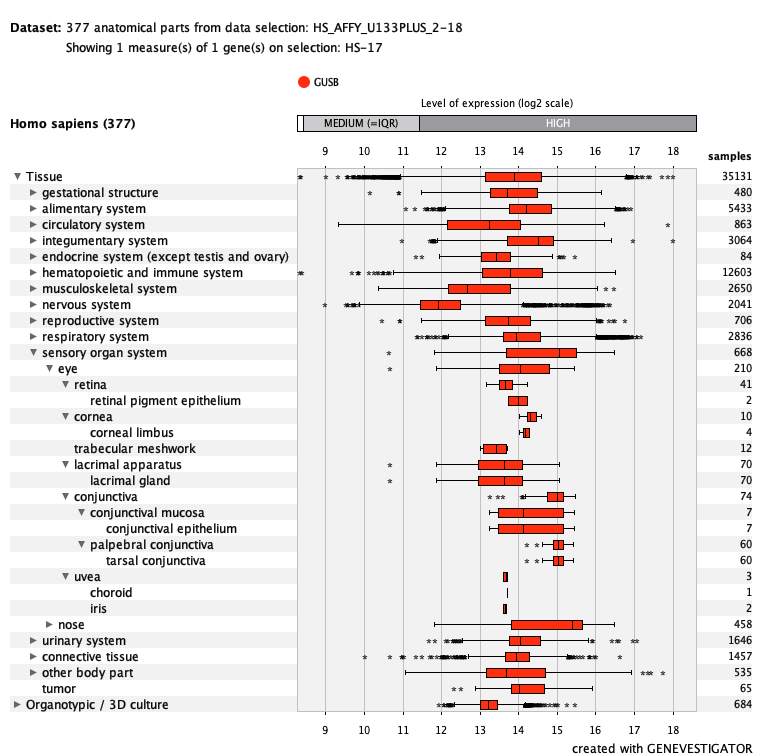
*

*HFM1*

*
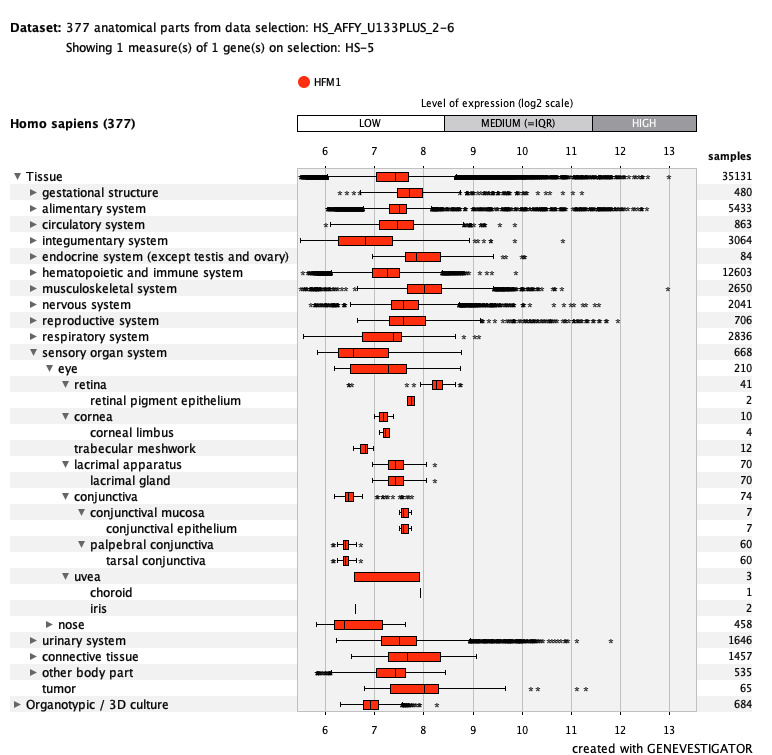
*

*HLA-B*

*
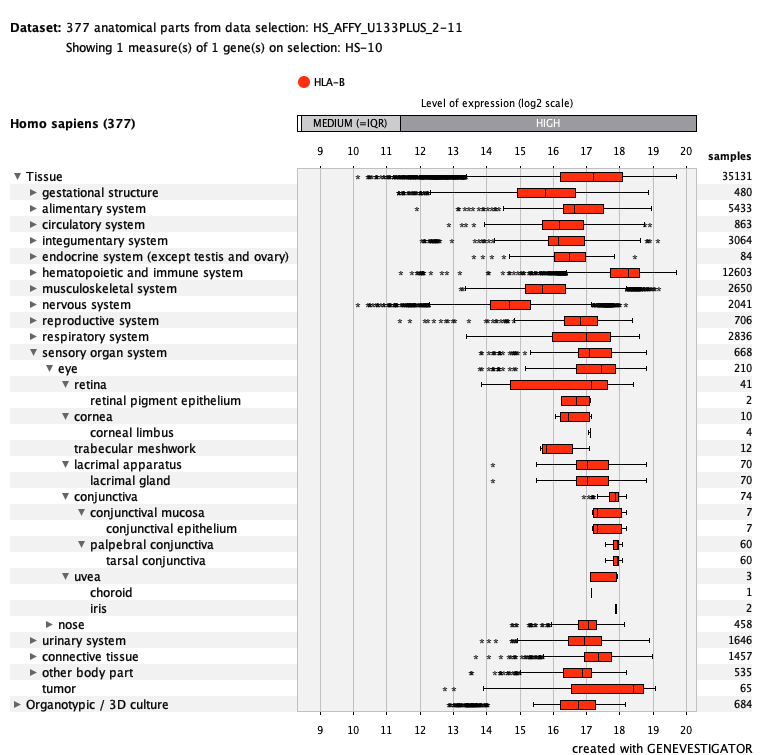
*

*IFI27*

*
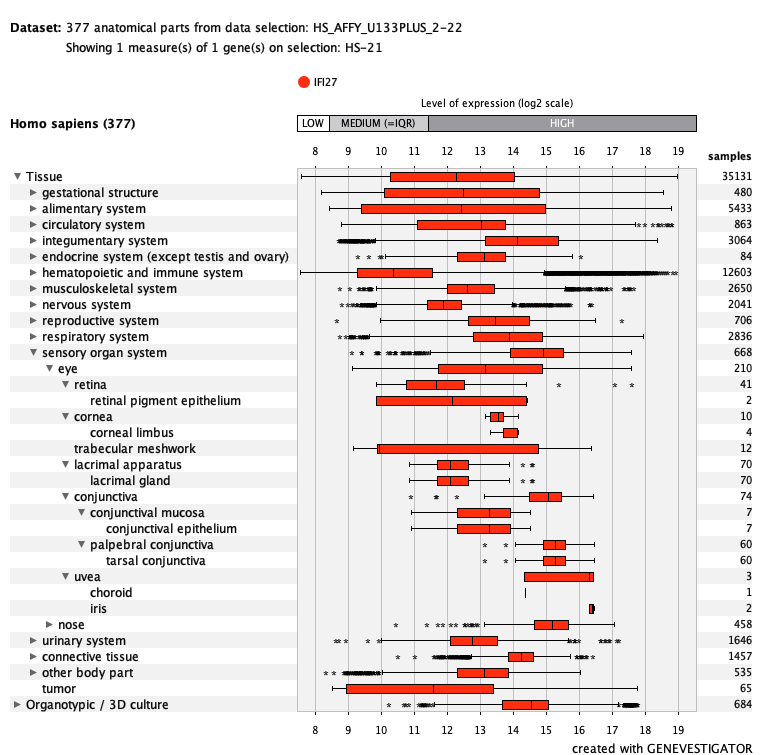
*

*ISY1-RAB43*

*
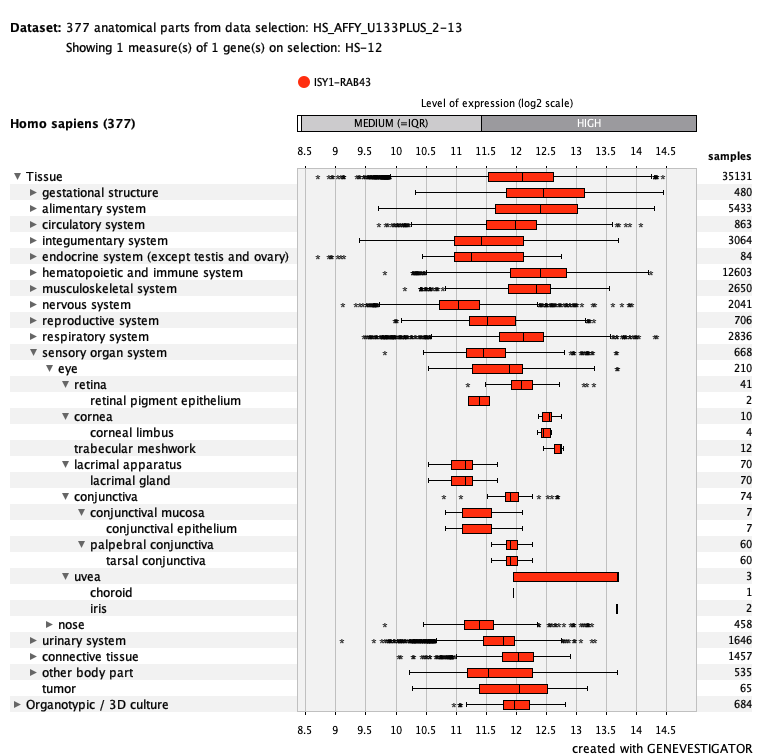
*

*KIF21A*

*
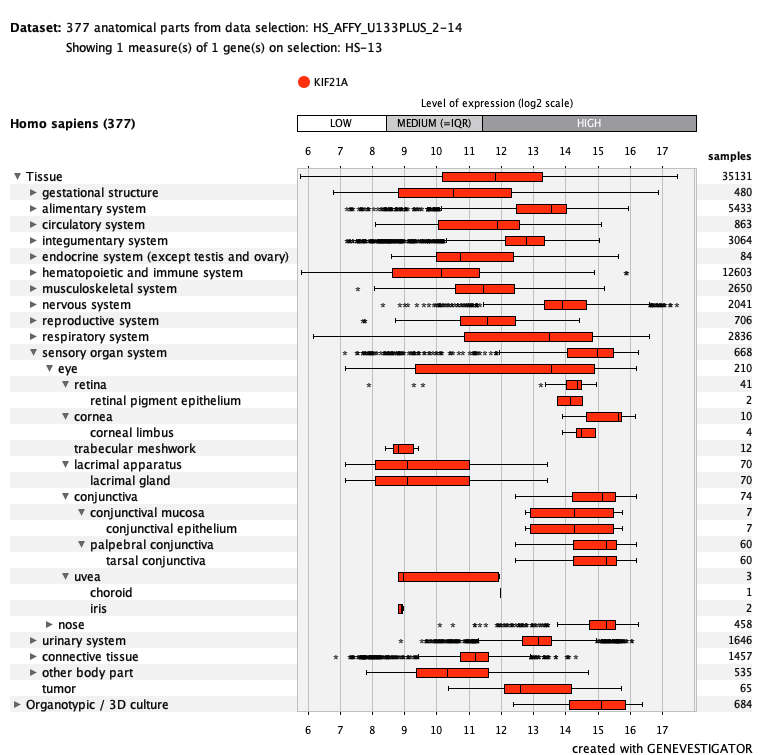
*

*LDB3*

*
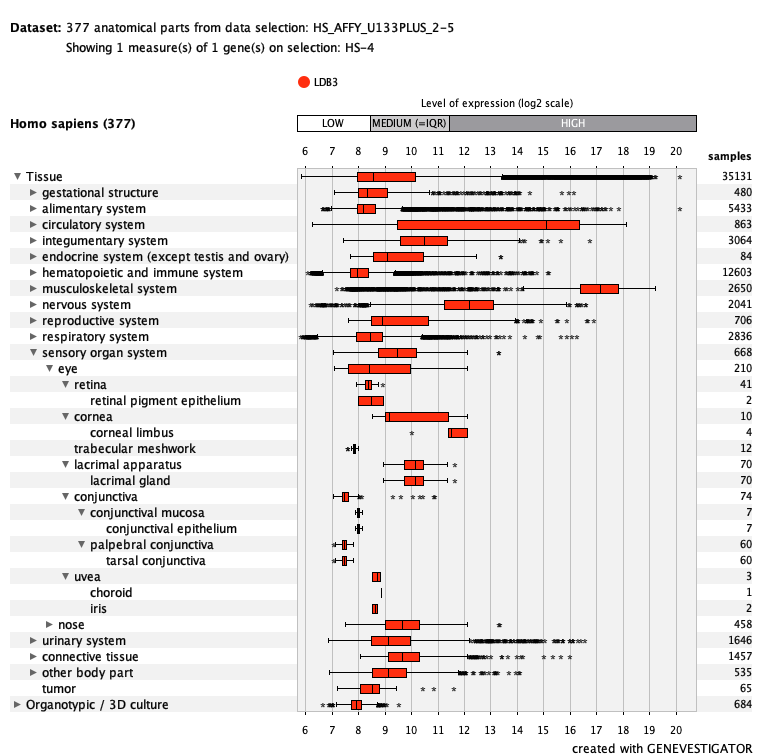
*

*MIR6891*

*
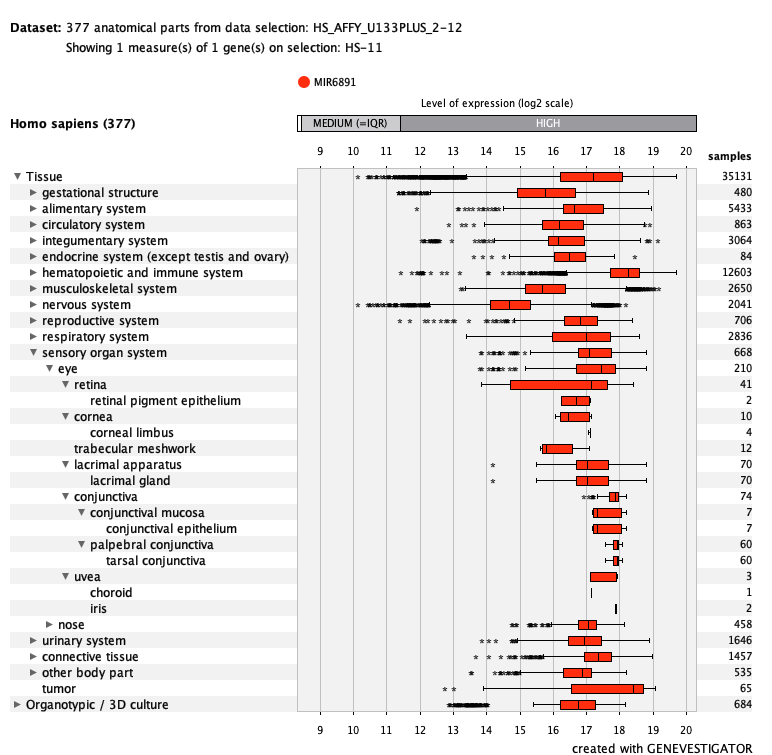
*

*MTOR*

*
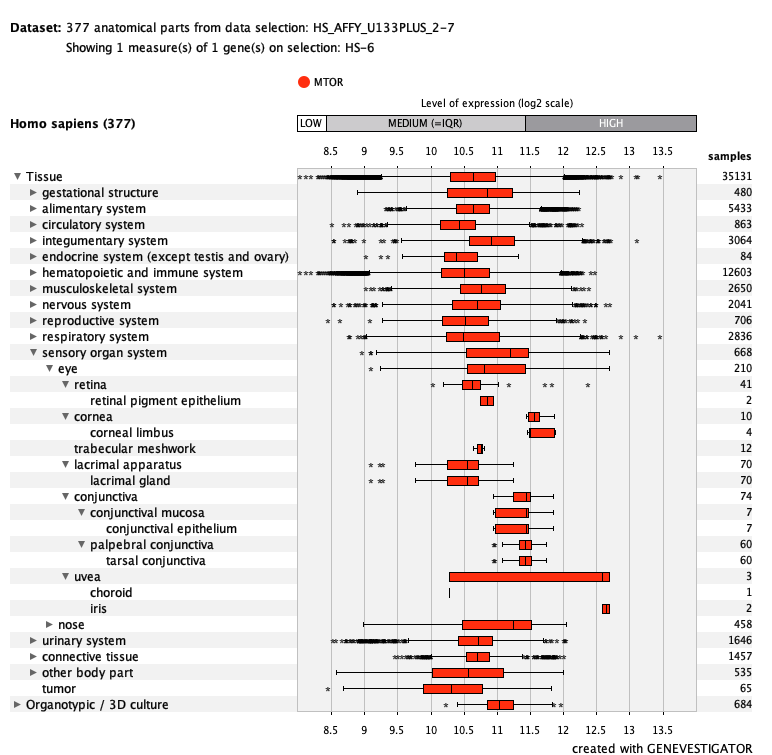
*


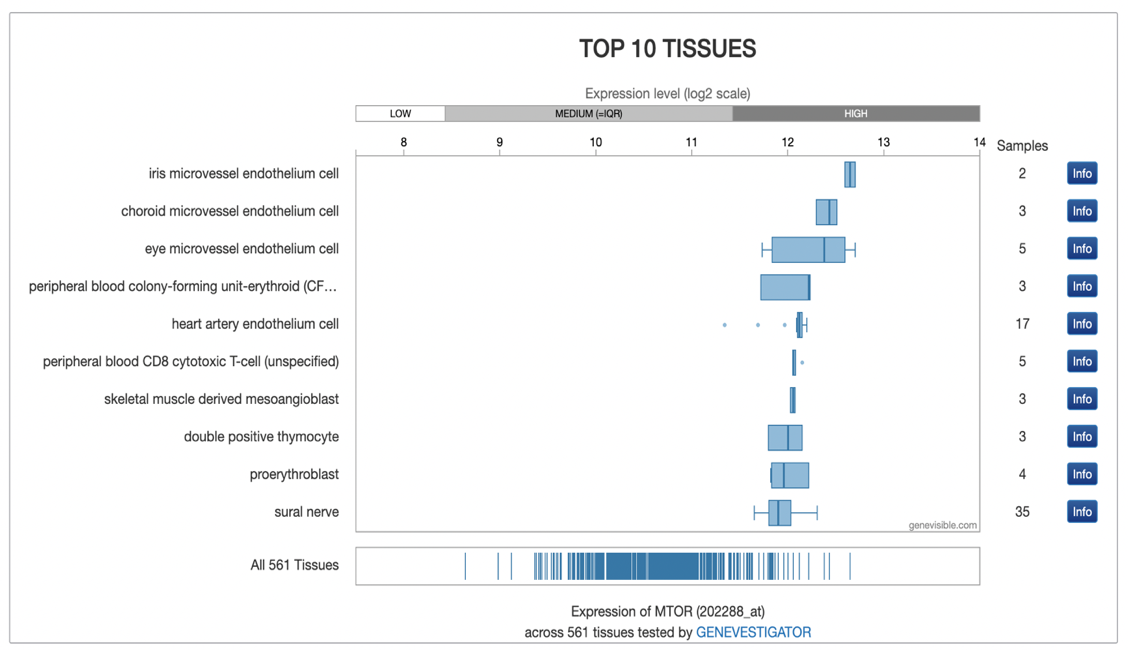


*MYOC*

*
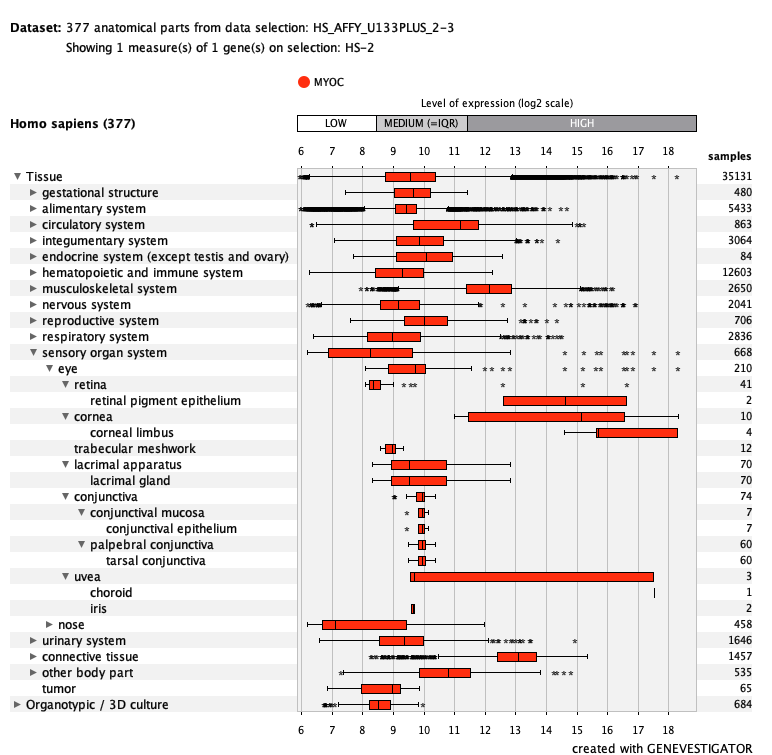
*

*NFXL1*

*
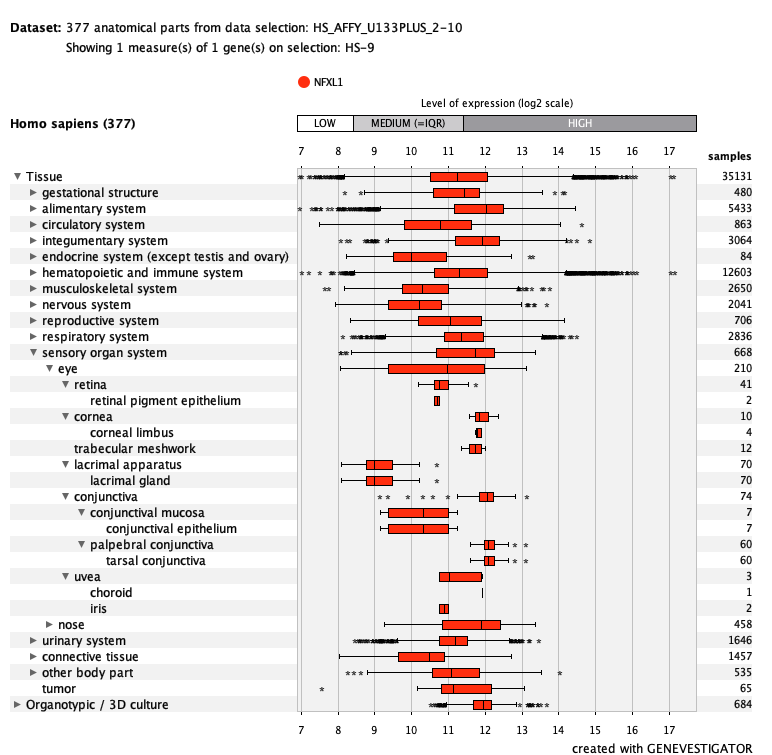
*

*PLAU*

*
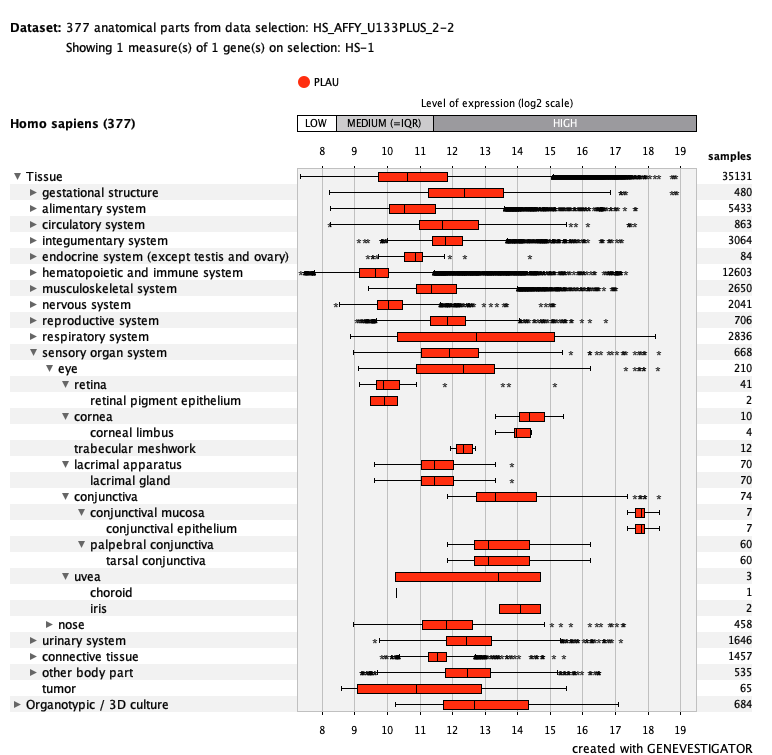
*

*PLK5*

*
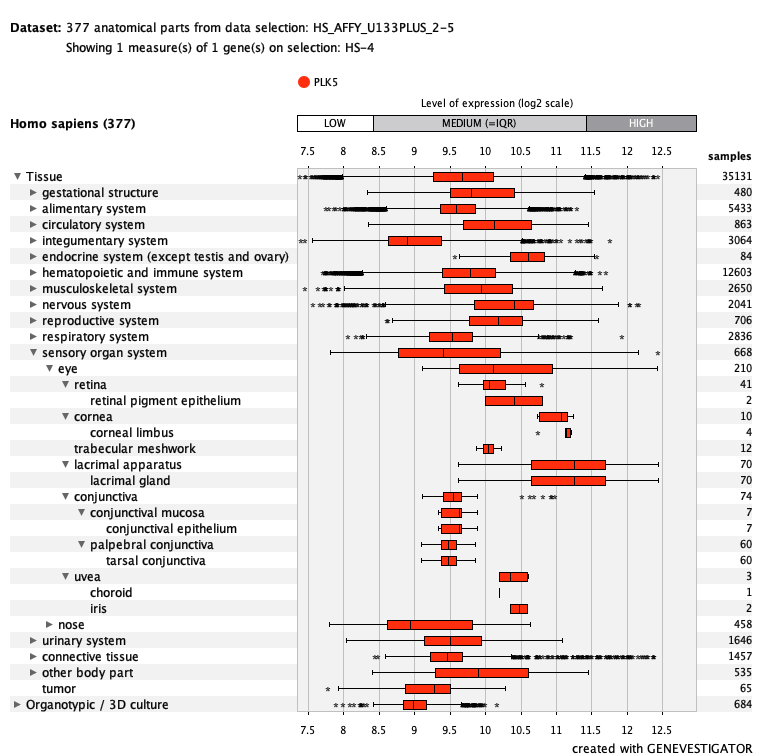
*

*PPIF*

*
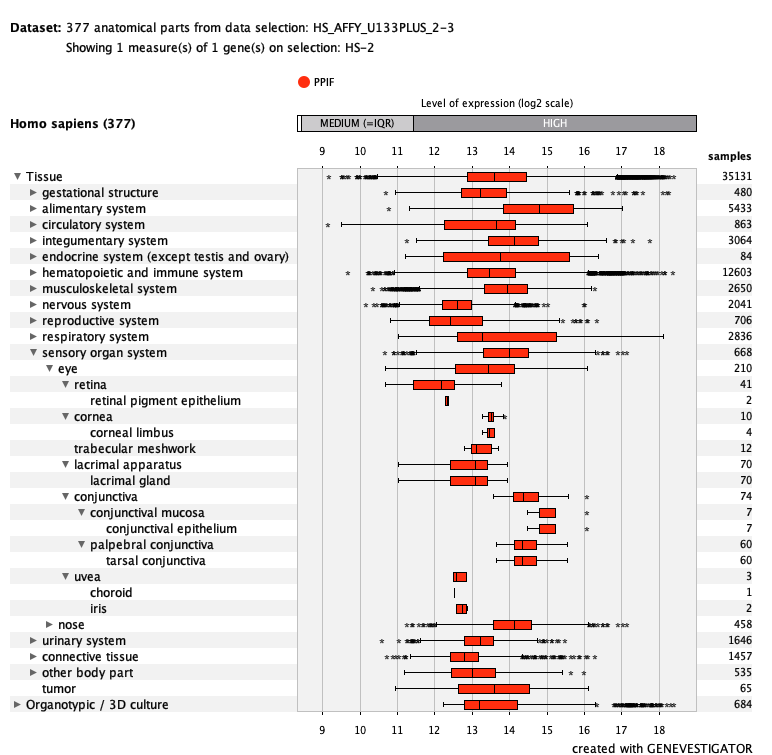
*

*PPM1J*

*
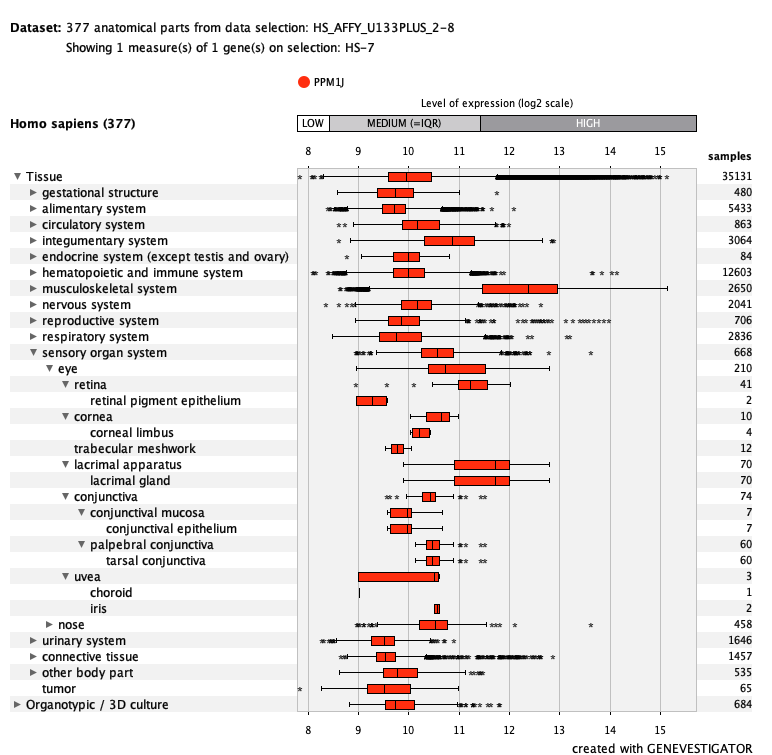
*

*PTPRB*

*
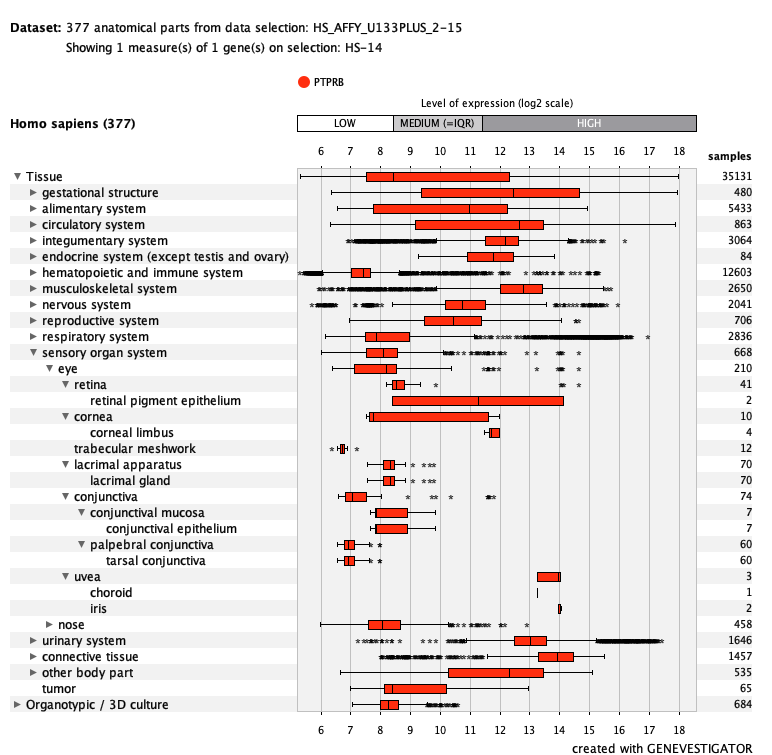
*


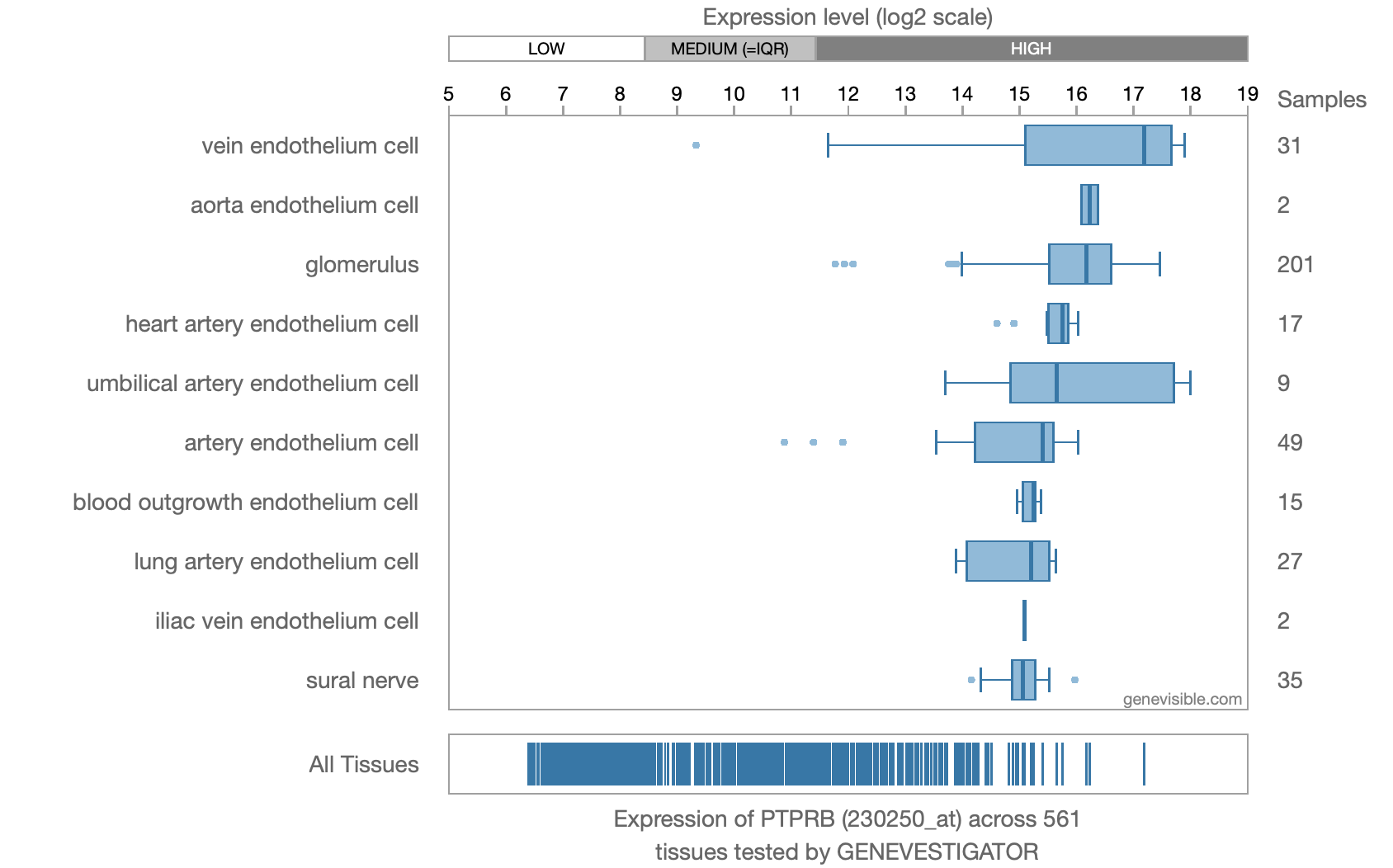


*RAB4B-EGLN2*

*
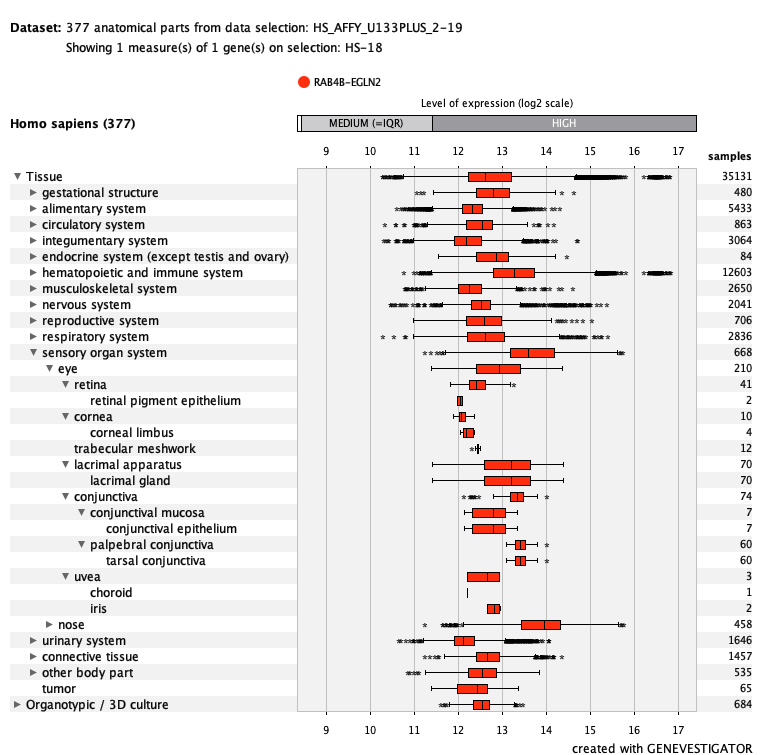
*

*RALYL*

*
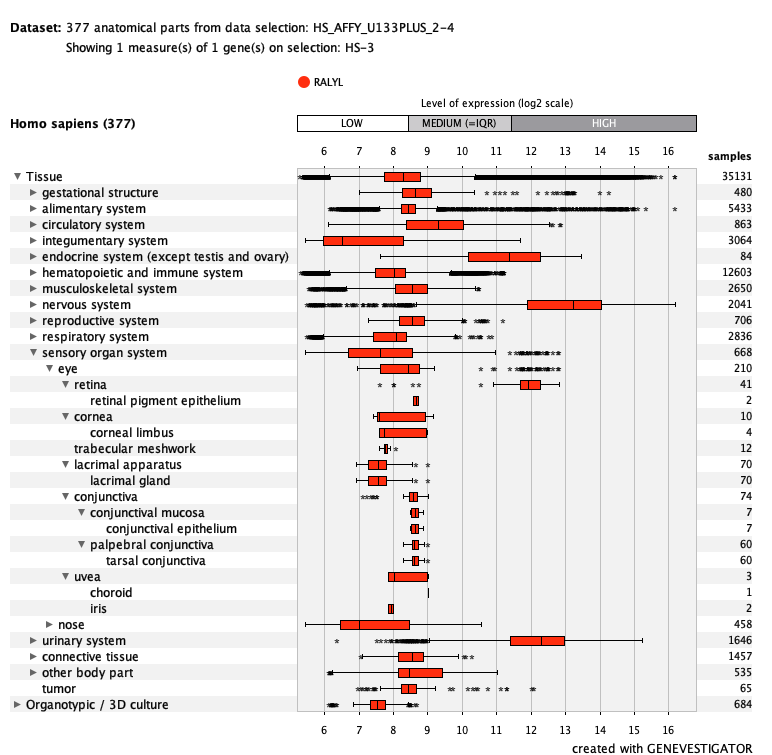
*

*RHOC*

*
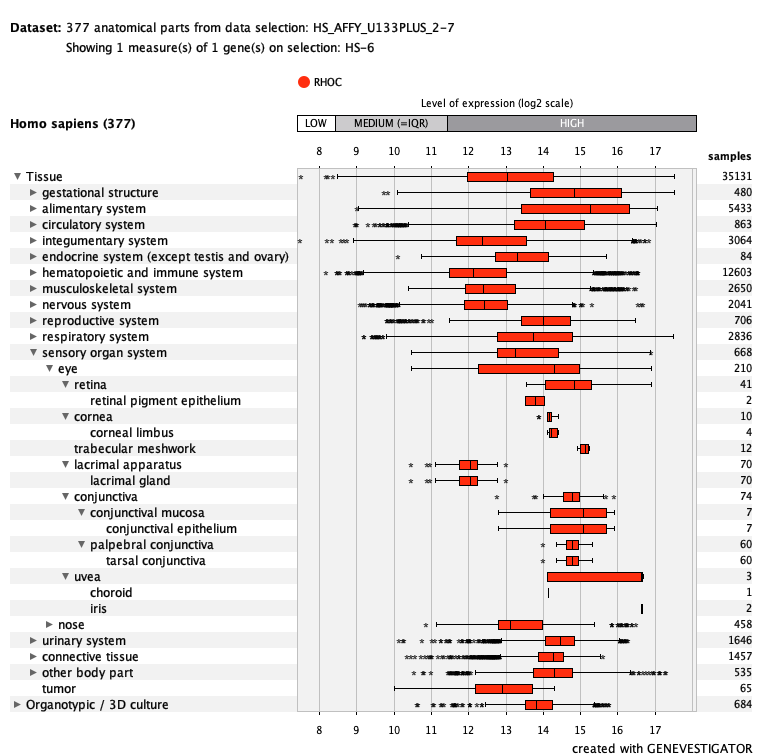
*

*RPL10A*

*
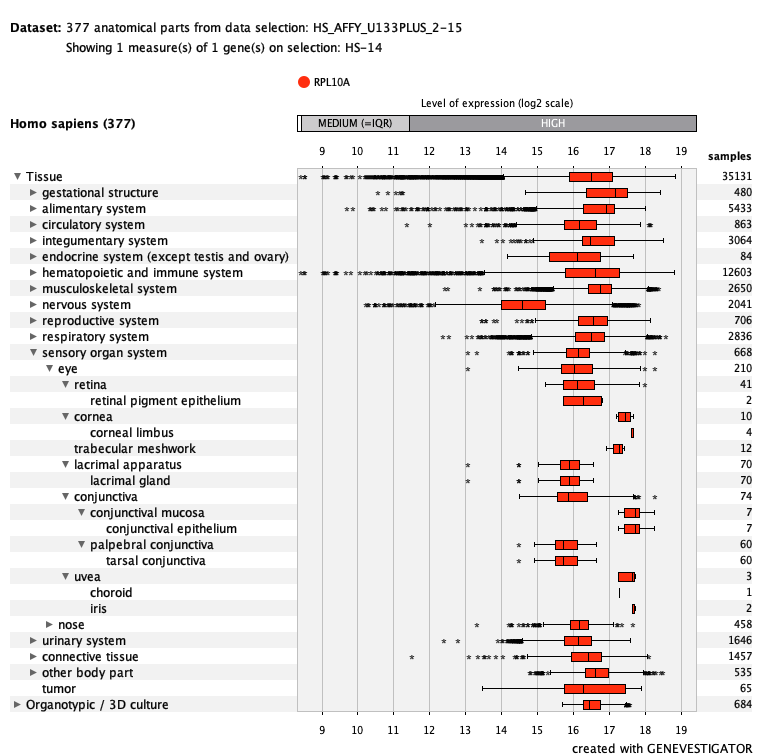
*

*RPL26L1*

*
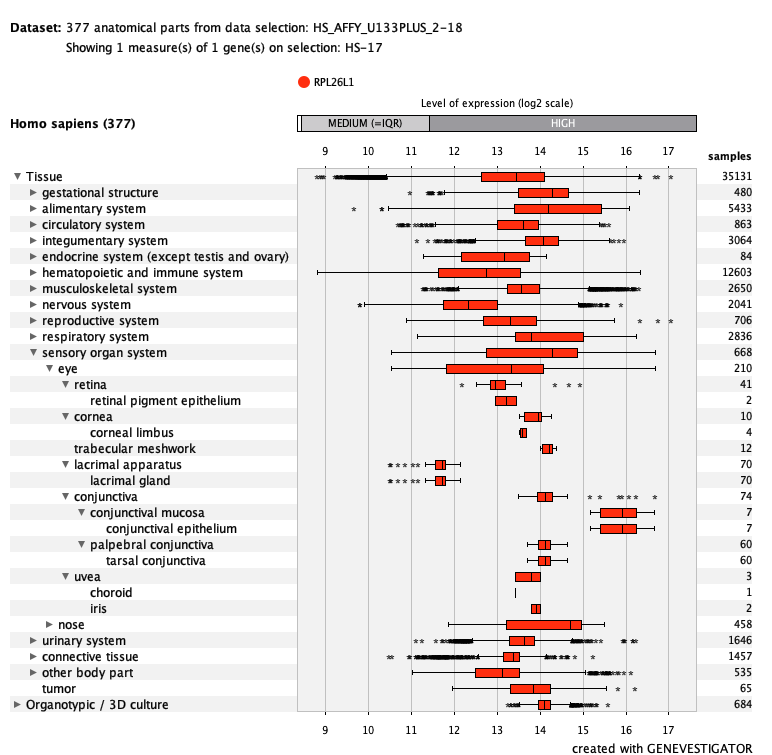
*

*SMG7*

*
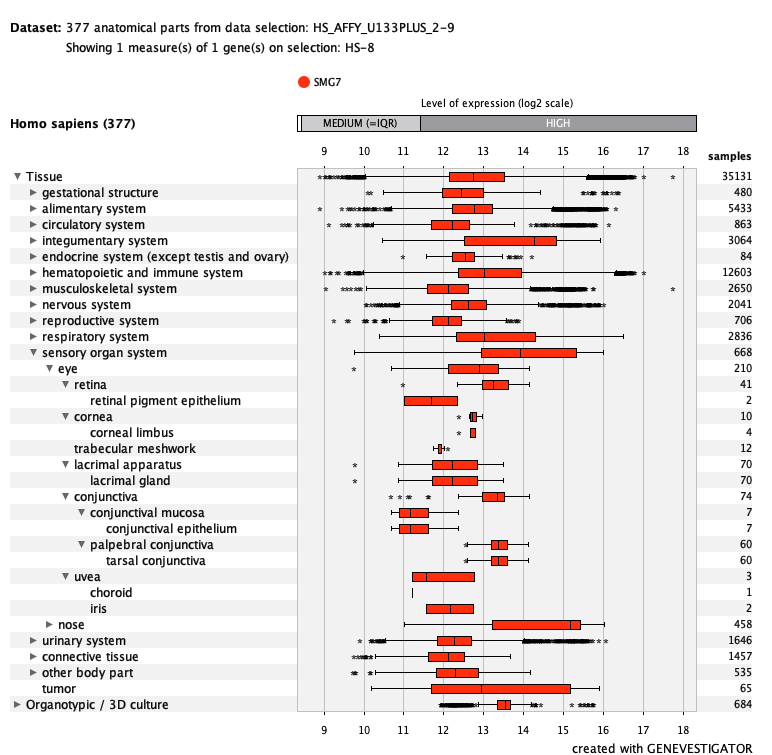
*

*SOS2*

*
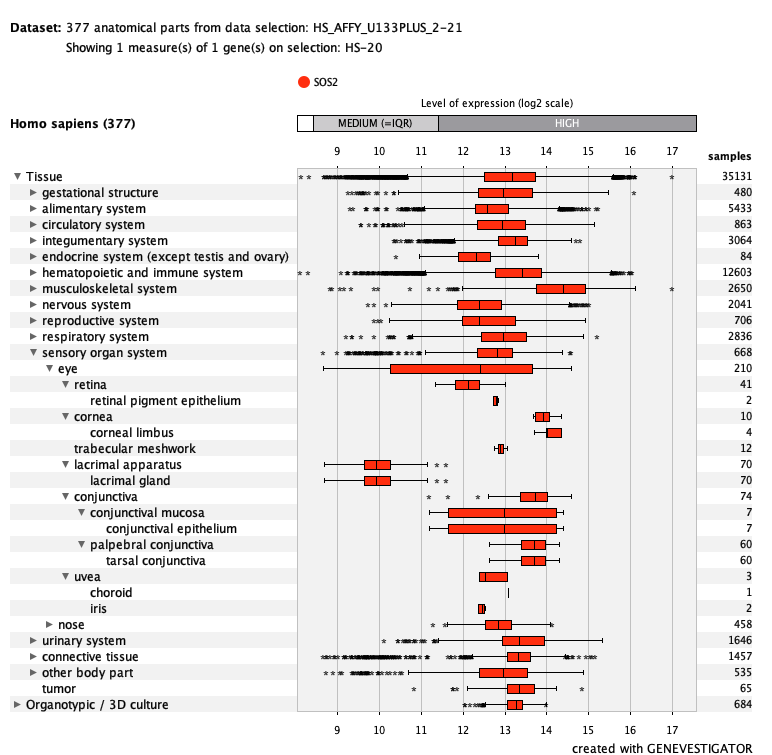
*

*SYNGR3*

*
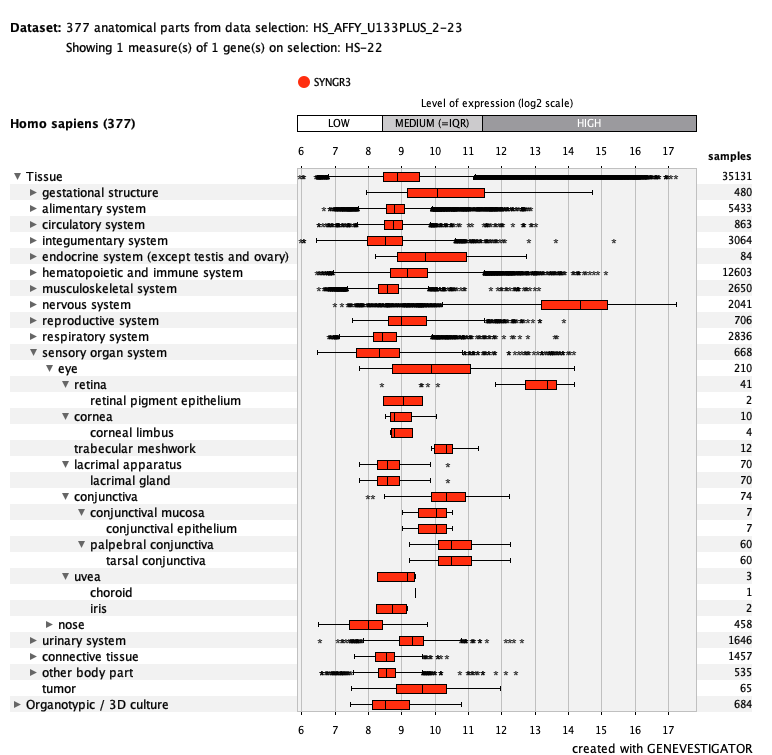
*

*TAF1B*

*
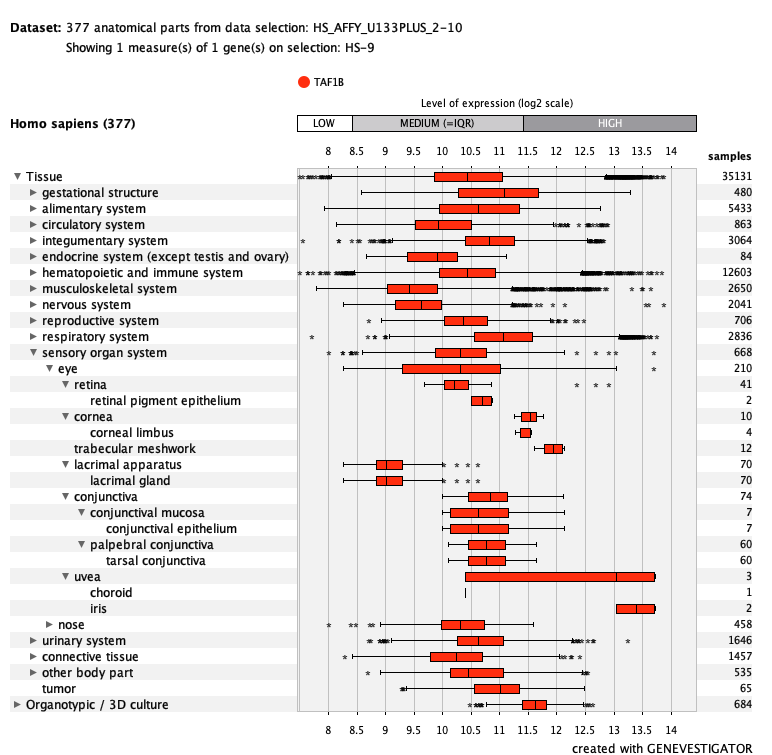
*

*TCP10L*

*
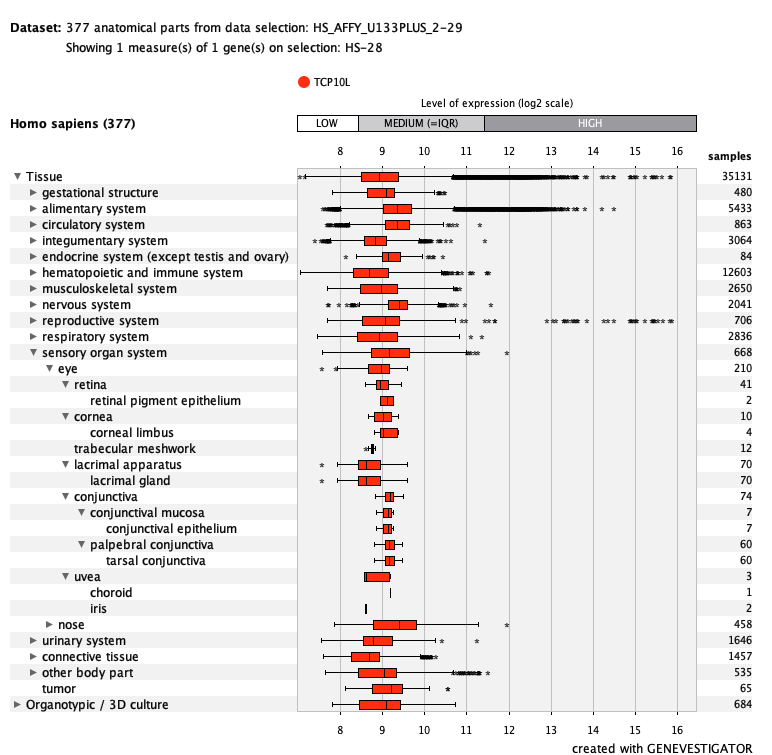
*

*TEAD3*

*
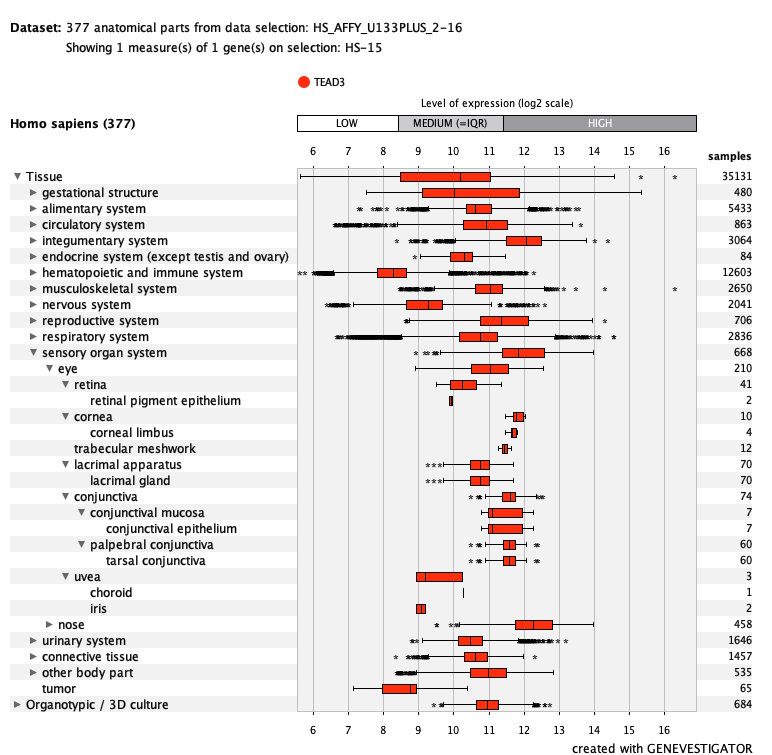
*

*UBN1*

*
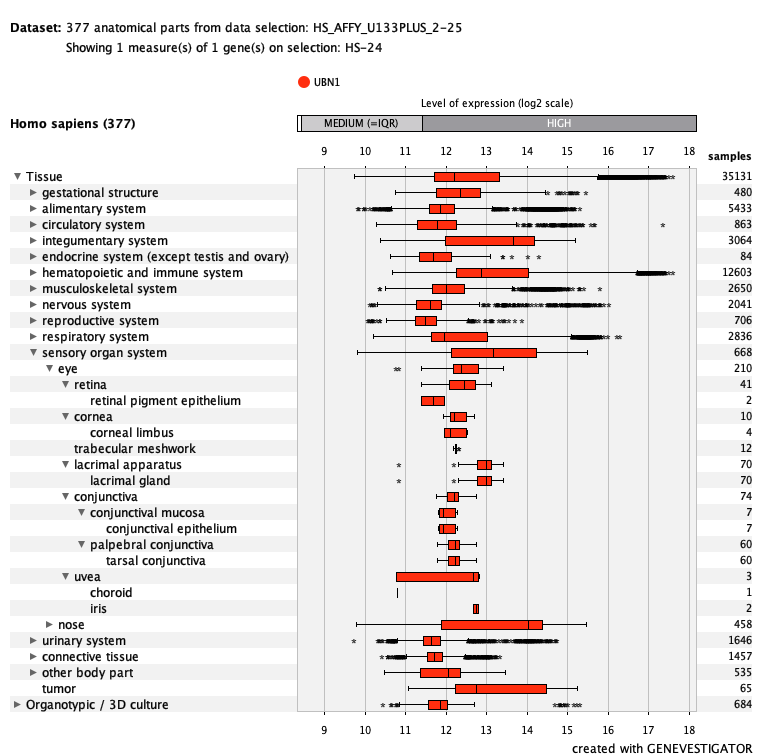
*

*VKORC1L1*

*
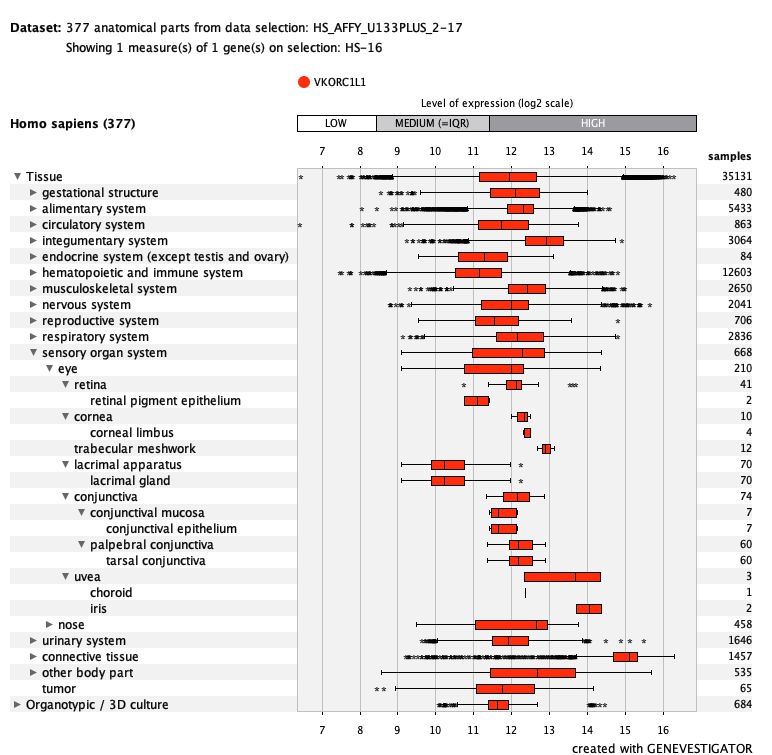
*

*ZNF598*

*
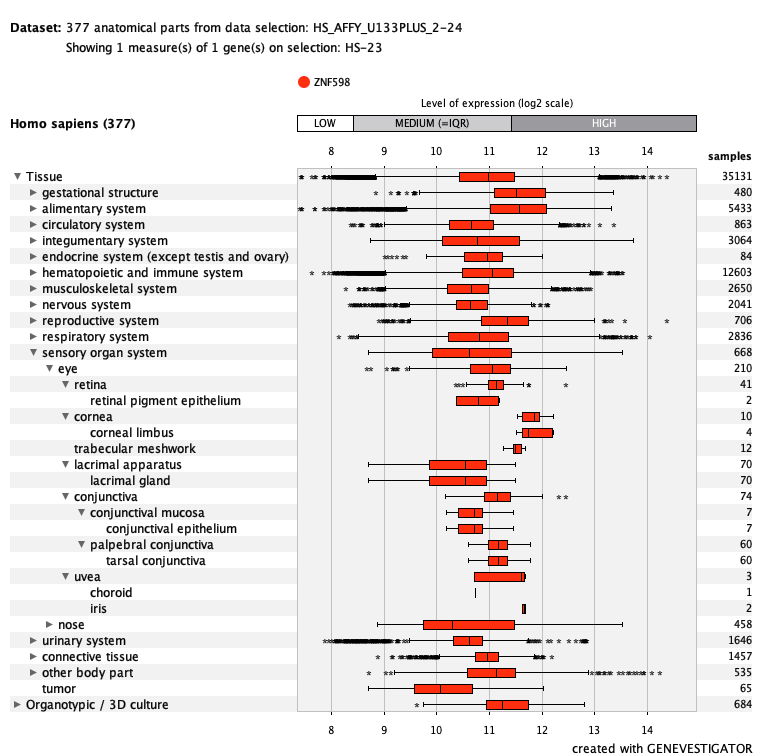
*

**Supplementary Figure 3. Single-cell RNA sequencing profiles from Broad Institute**

We used the Cell atlas of the human ocular anterior segment^2^ (queried through the Broad Institute Single Cell Portal, <https://singlecell.broadinstitute.org/single_cell/study/SCP1841/>). Single cell RNA sequencing profiles are listed alphabetically according to gene name.

Abbreviations: CC_VenEndo, Collector channel vascular endothelium; Ciliary_Muscle, Ciliary muscle; Conj_Epi-Basal, Conjunctiva epithelial basal; Conj_Epi-Superficial, Conjunctiva epithelial superficial; Conj_Epi-Wing, Conjunctiva epithelial wing; Conj_Melanocyte, Conjunctiva melanocyte; FibroX, Scleral fibroblast; Goblet, Goblet; K_Endo, Corneal endothelium; K_Epi-Basal, Corneal epithelium basal; K_Epi-Superficial, Corneal epithelial superficial; K_Epi-Wing, Corneal epithelial wing; K_Fibro, Corneal fibroblast; Limbal_Epi-Basal, Limbal epithelium basal; Limbal_Epi-Superficial, Limbal epithelial superficial; Limbal_Epi-Wing, Limbal epithelial wing; Lymphatic_Endo, Lymphatic endothelium; Schlemm_Endo, Schlemm endothelium; Sclera_Fibro, Sclera fibroblast; TM_Fibro, Trabecular meshwork fibroblast; Uveal_Fibro, Uveal fibroblast; VascEndo, Vascular endothelium.

*AAK1*

*
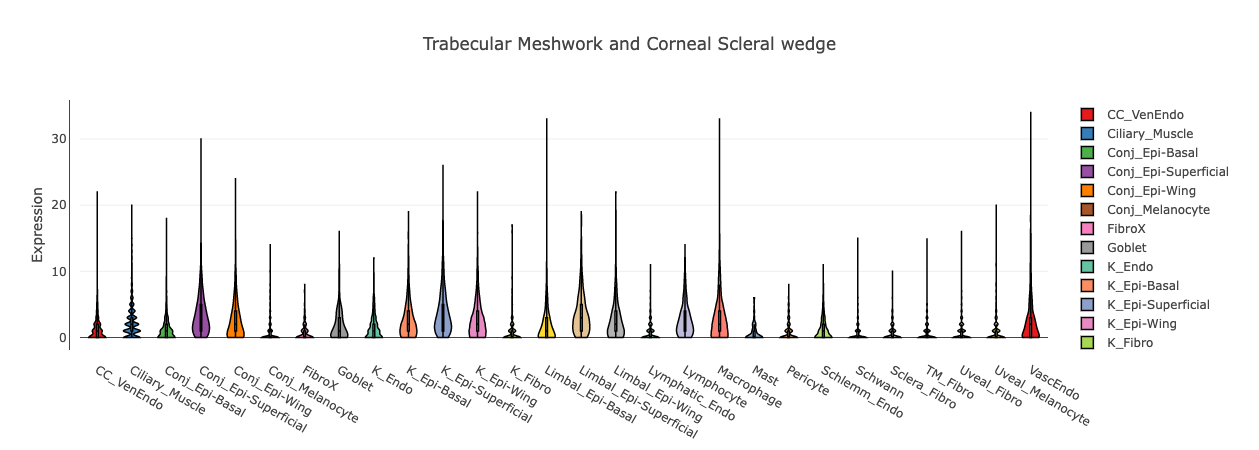
*

*ABI3BP*

*
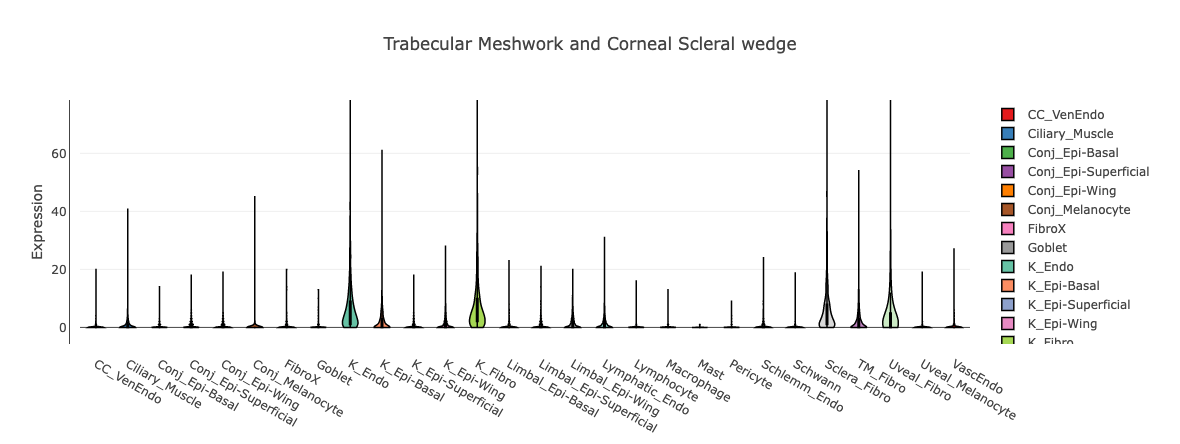
*

*ACAD10*

*
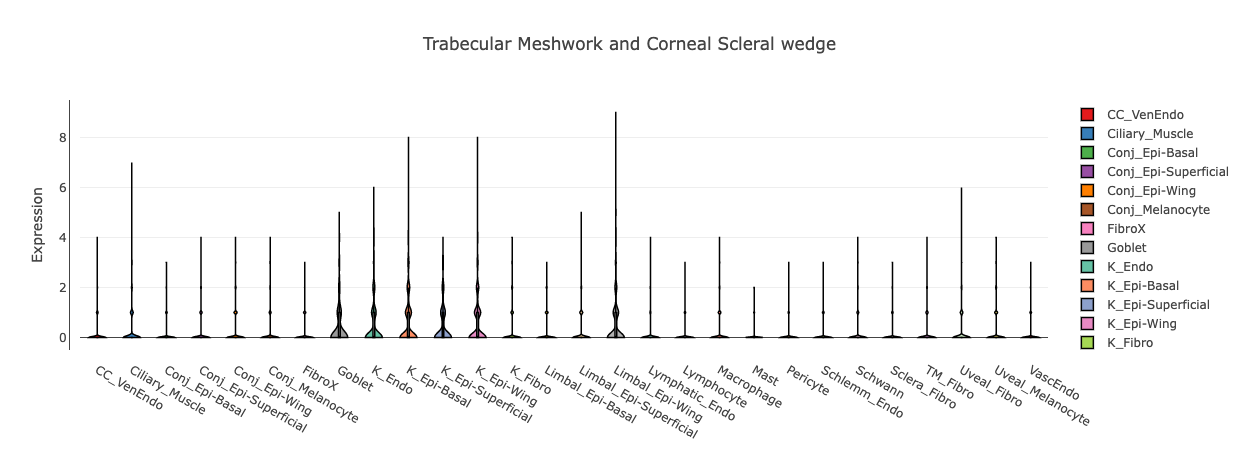
*

*ADRB1*

*
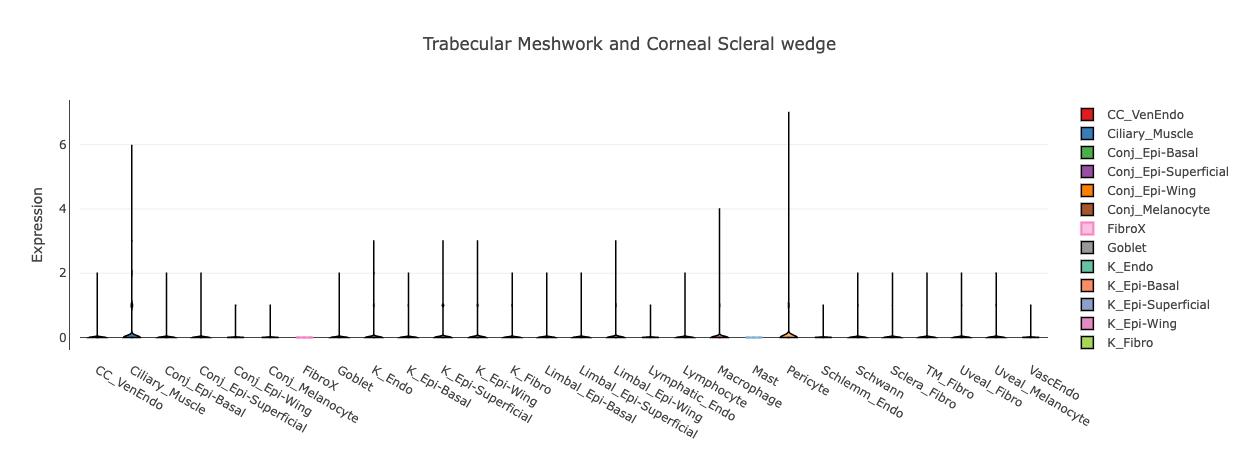
*

*ADSS2*

*
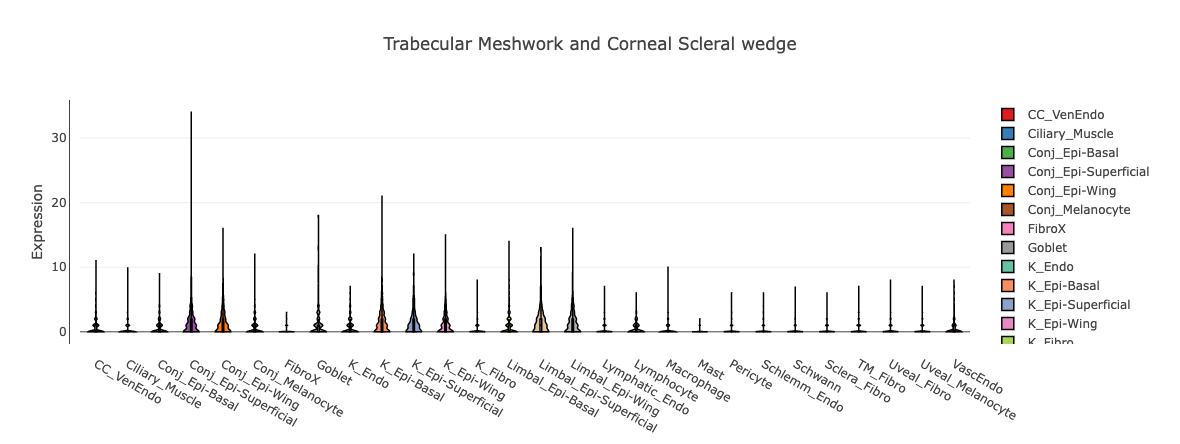
*

*ANGPTL7*

*
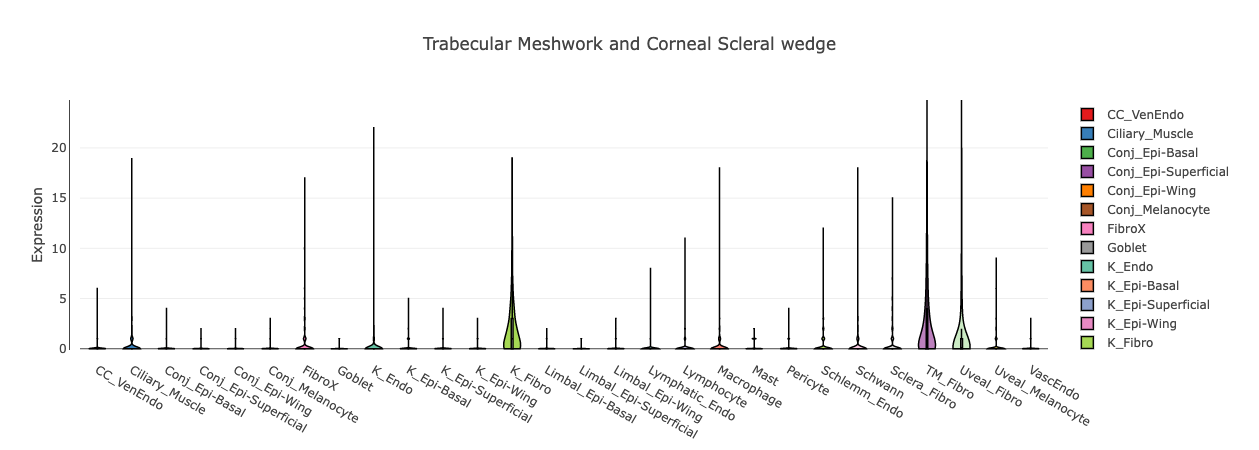
*

*BOD1L1*


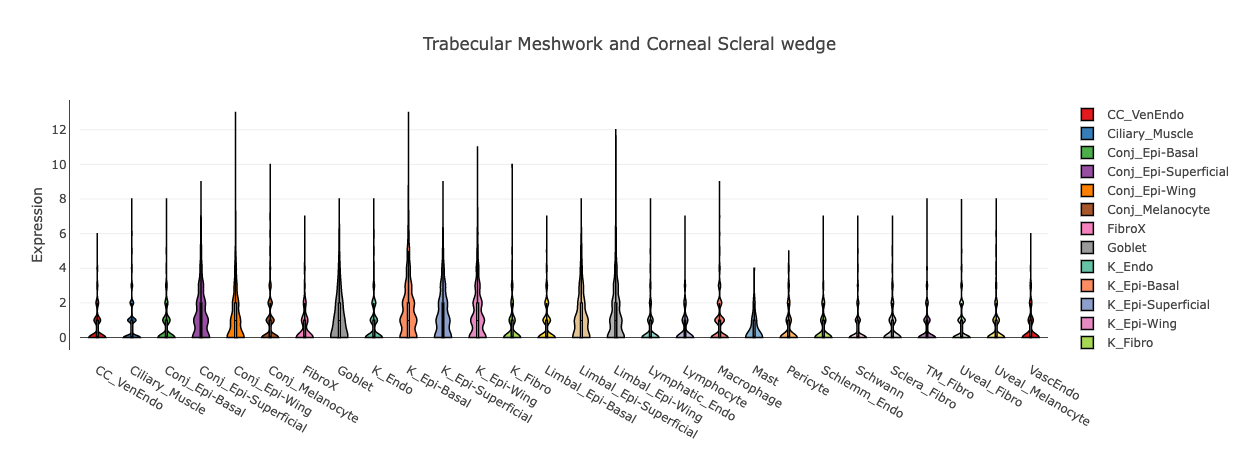


*CDCA8*

*
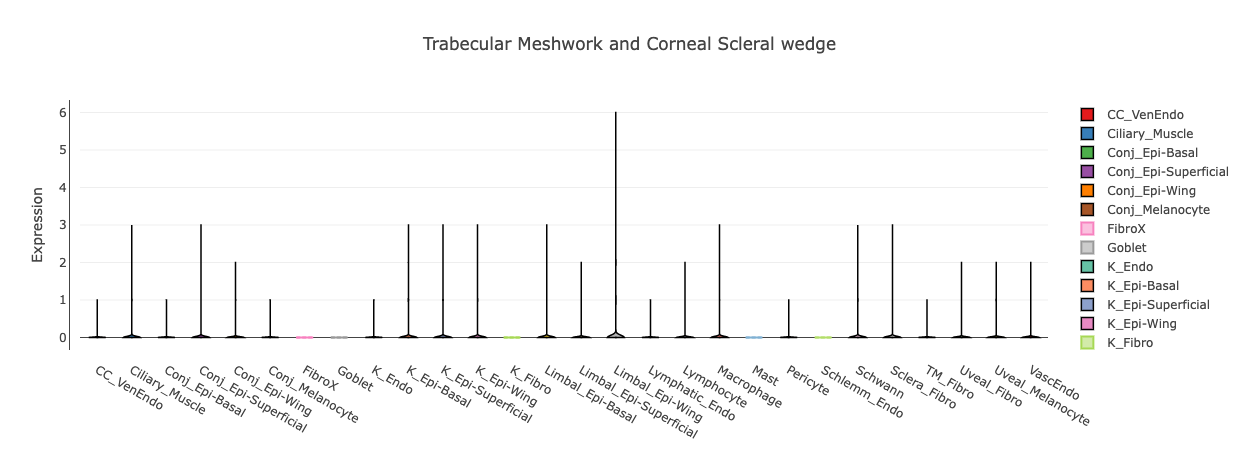
*

*CDK11A*

*
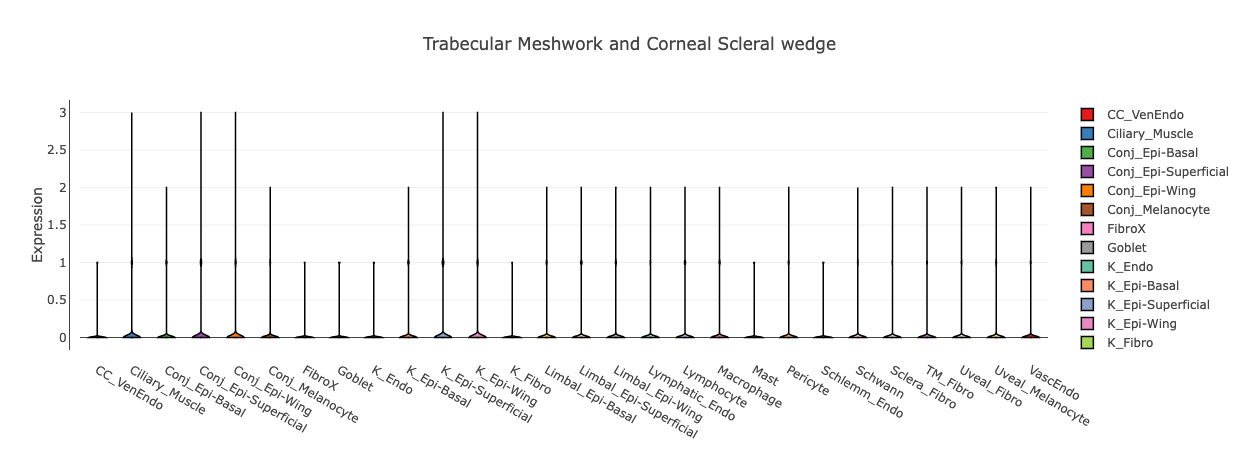
*

*CFAP298-TCP10L*

*
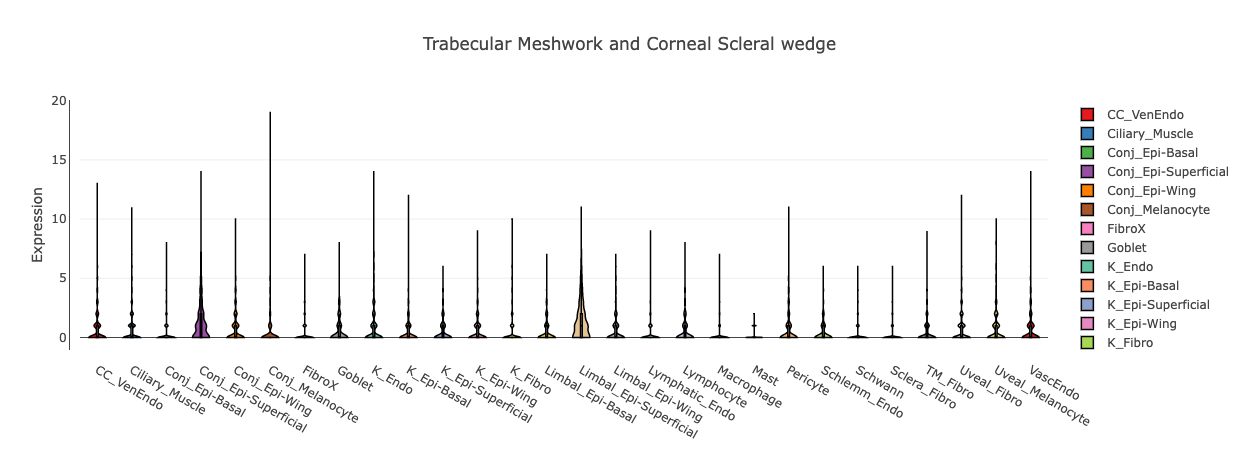
*

*DACT3*

*
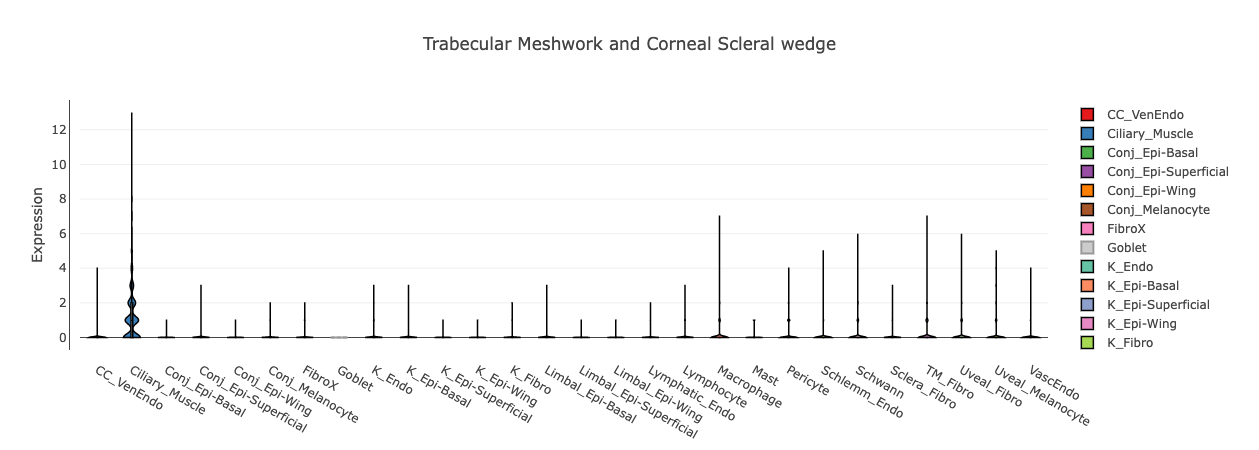
*

*DNTT*

*
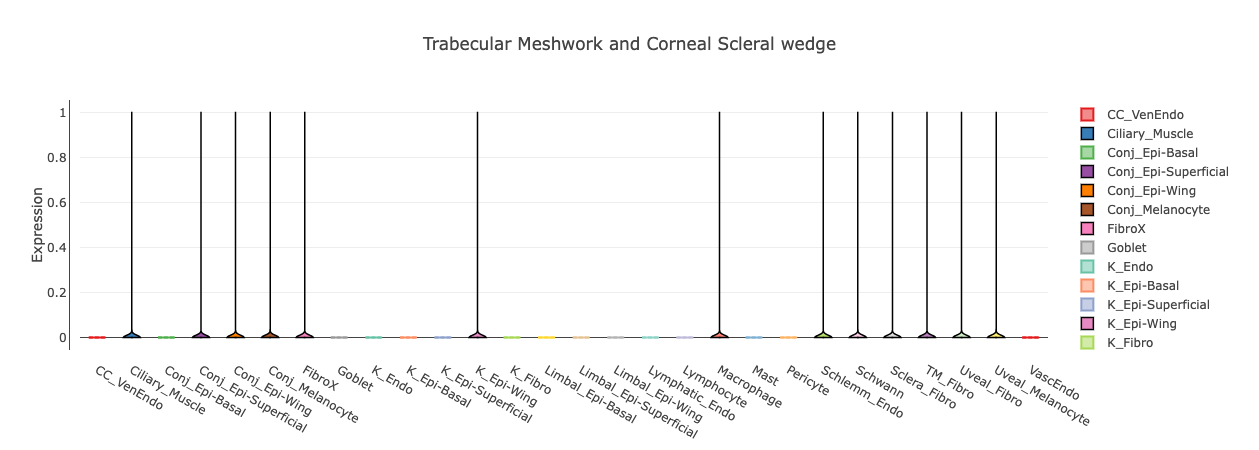
*

*DOK4*

*
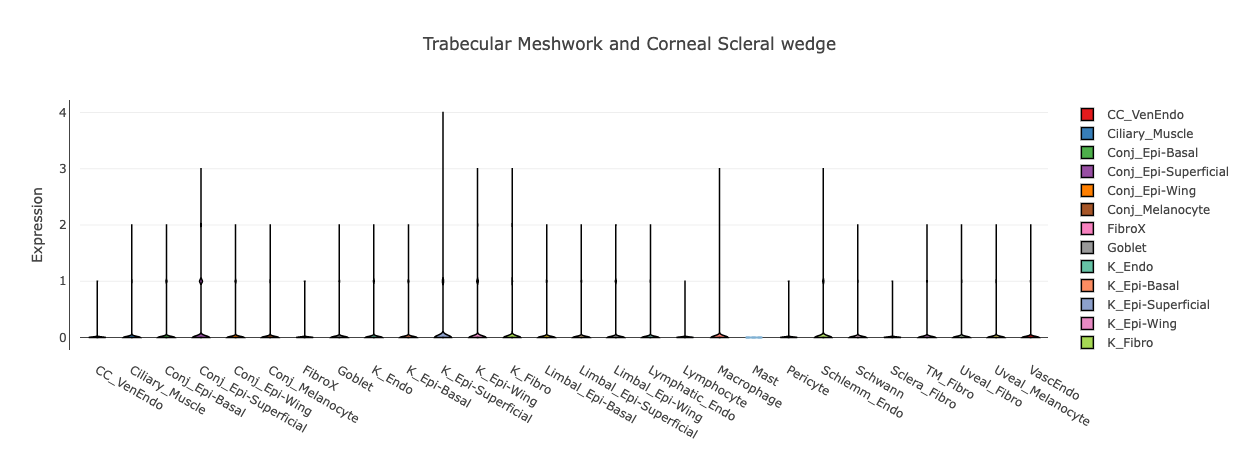
*

*DPF3*

*
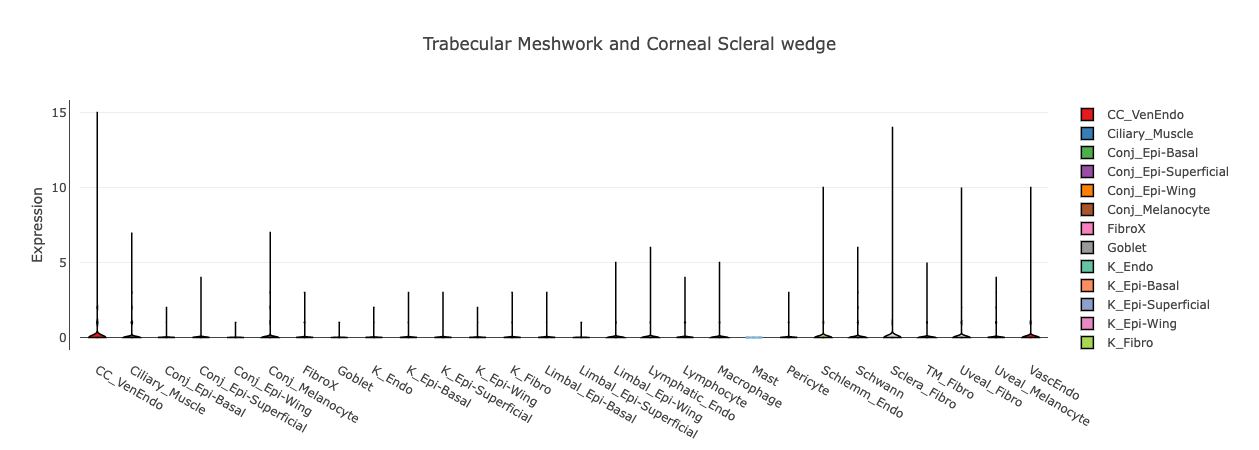
*

*EHMT1*

*
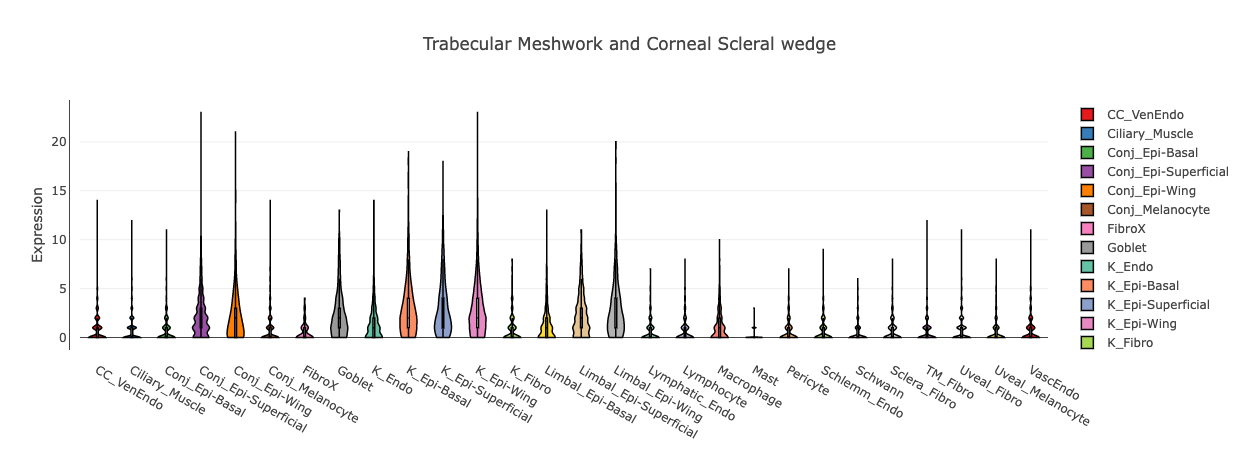
*

*EVA1C*

*
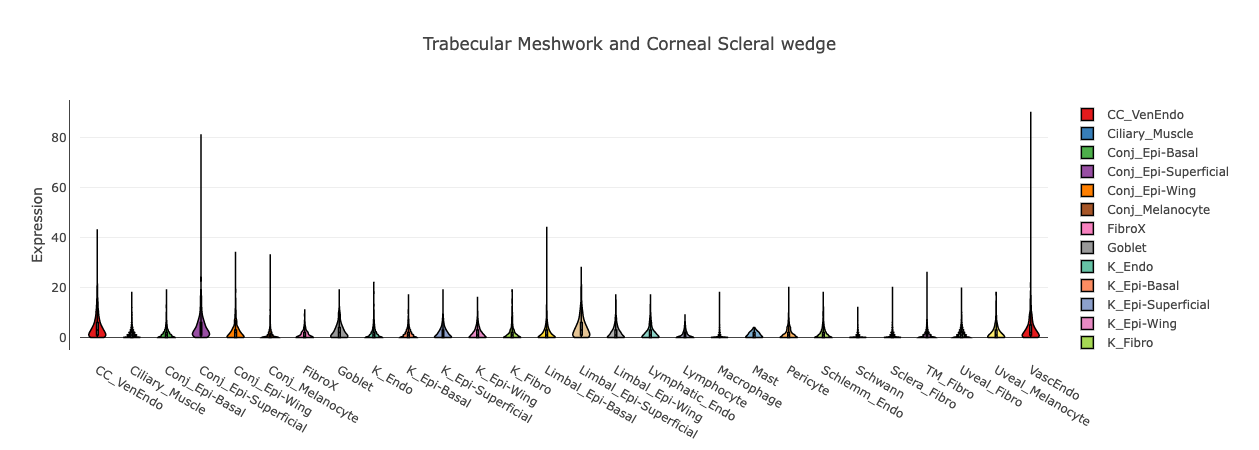
*

*FOXD1*

*
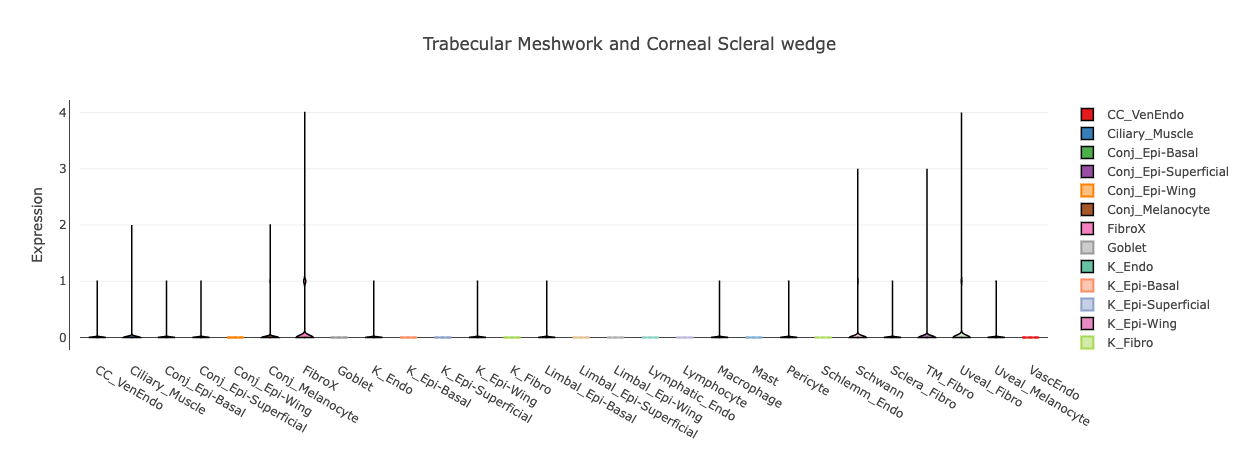
*

*GUSB*

*
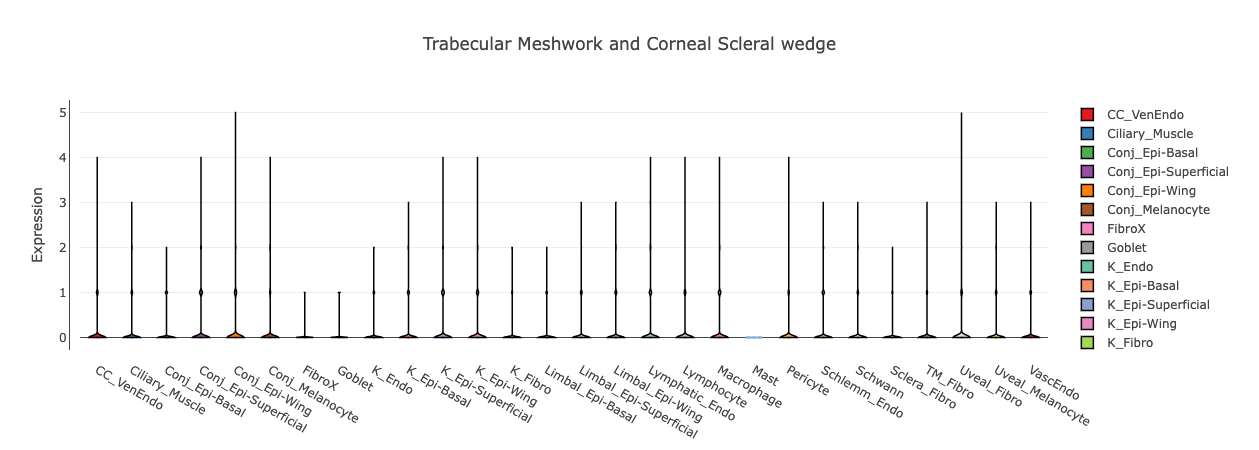
*

*HFM1*

*
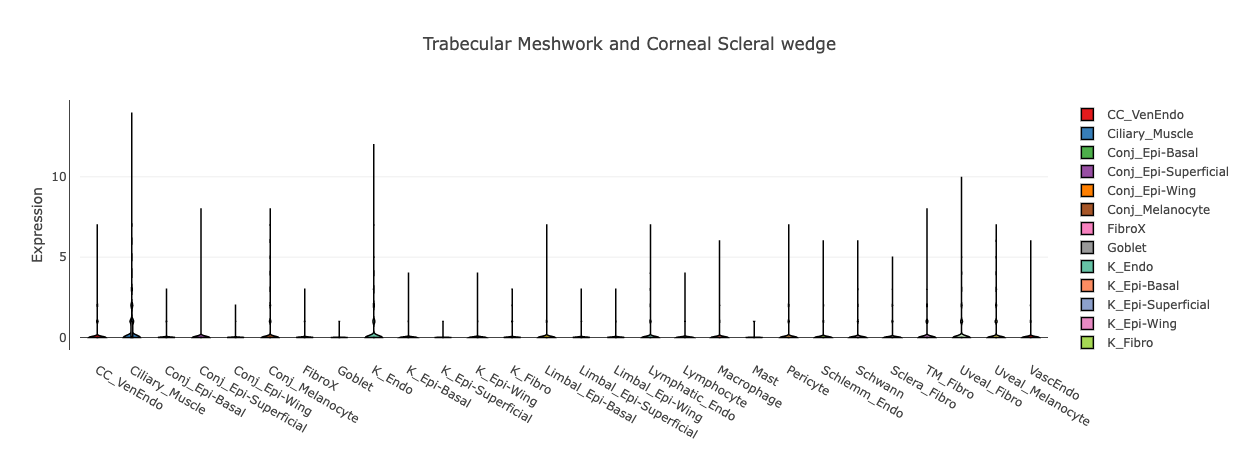
*

*HLA-B*

*
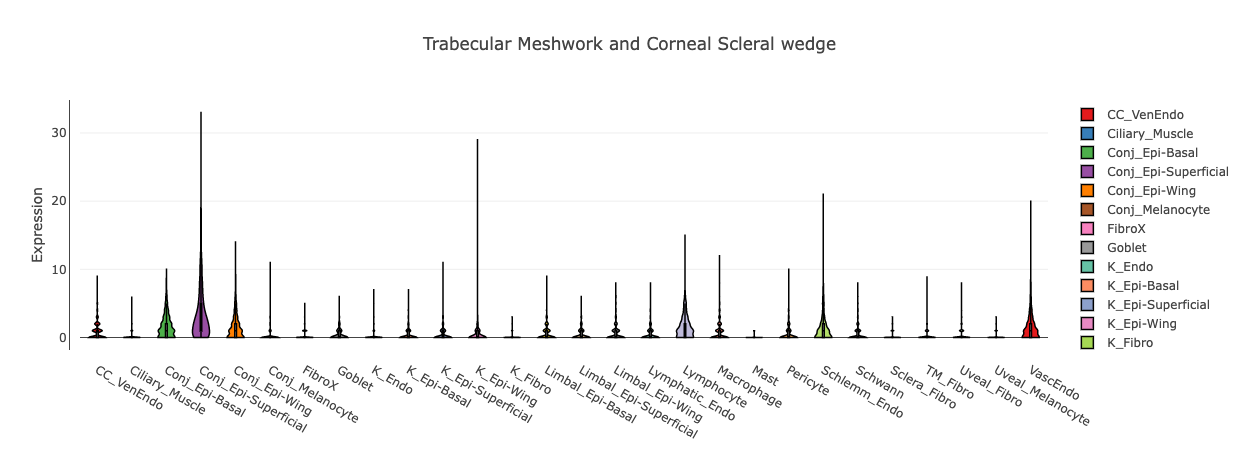
*

*IFI27*

*
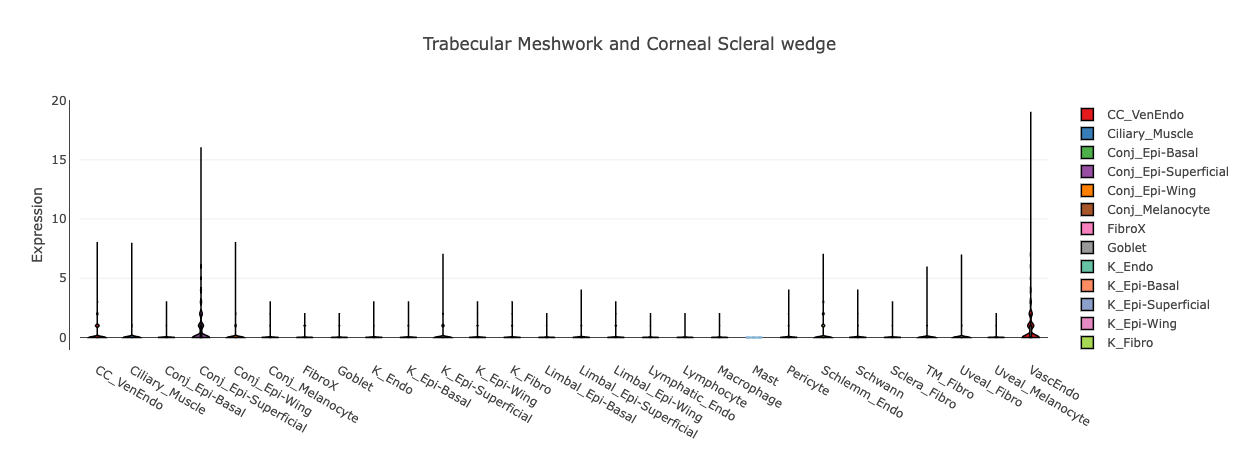
*

*ISY1-RAB43*

*
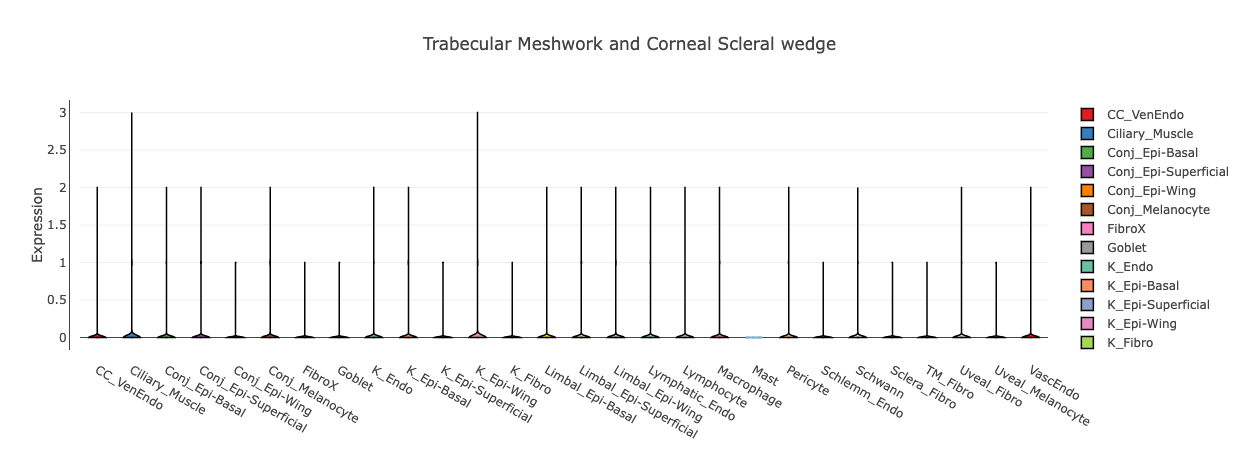
*

*KIF21A*

*
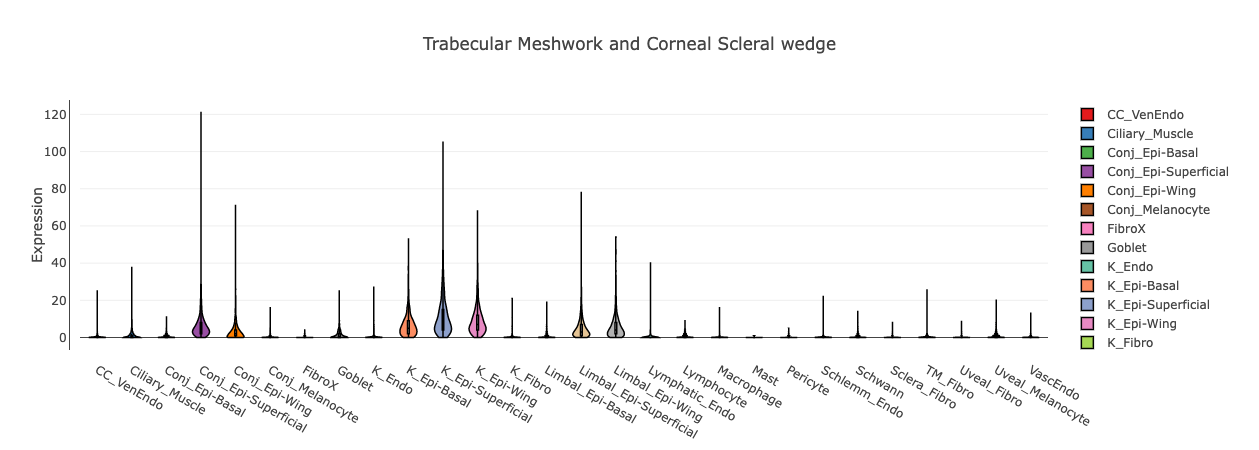
*

*LDB3*

*
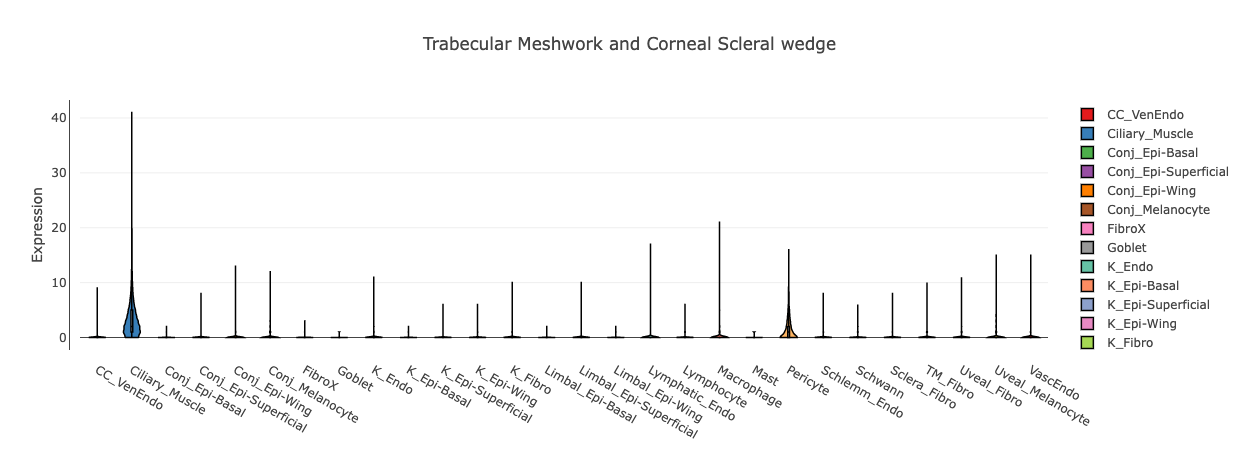
*

*MTOR*

*
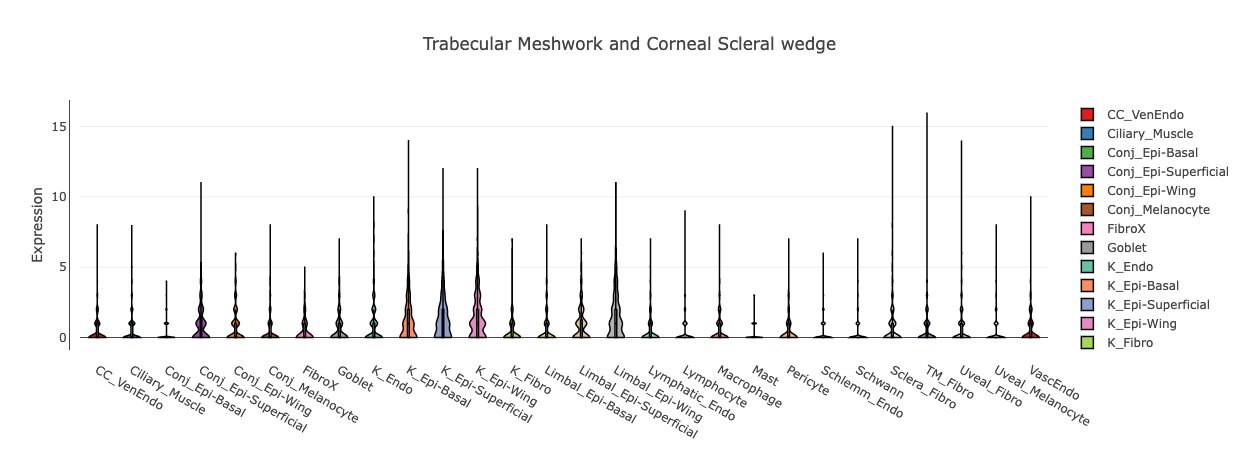
*

*MYOC*

*
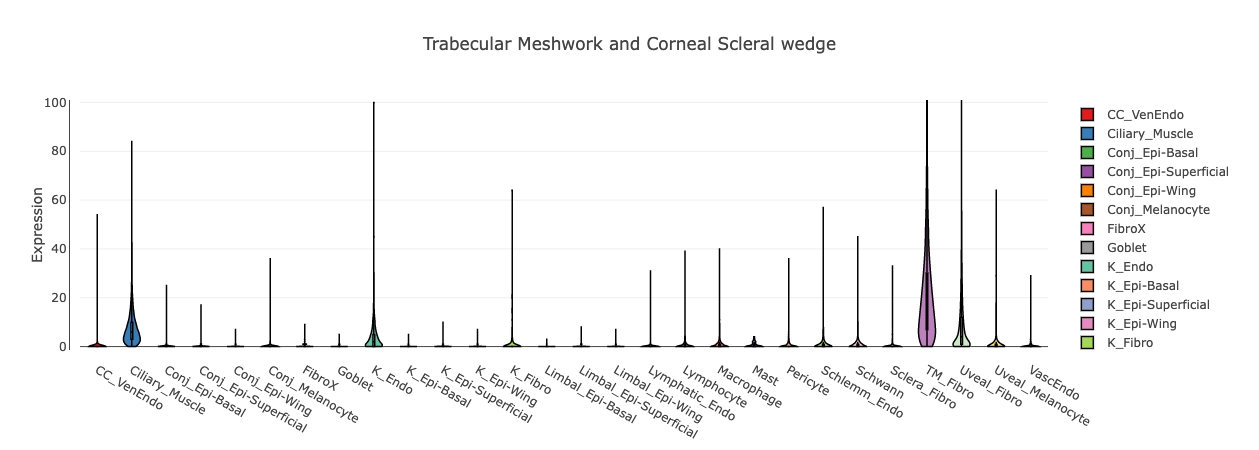
*

*NFXL1*

*
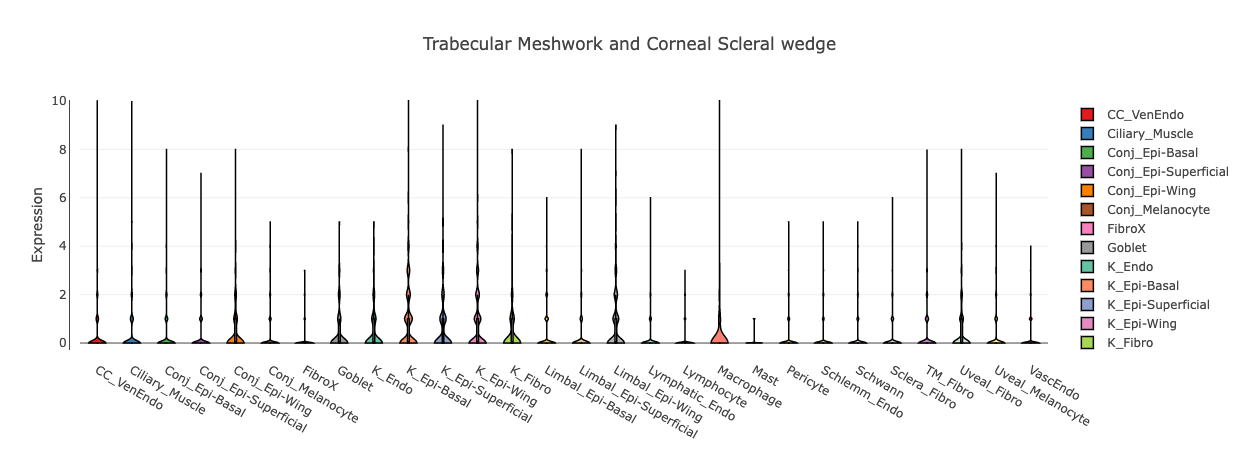
*

*PLAU*

*
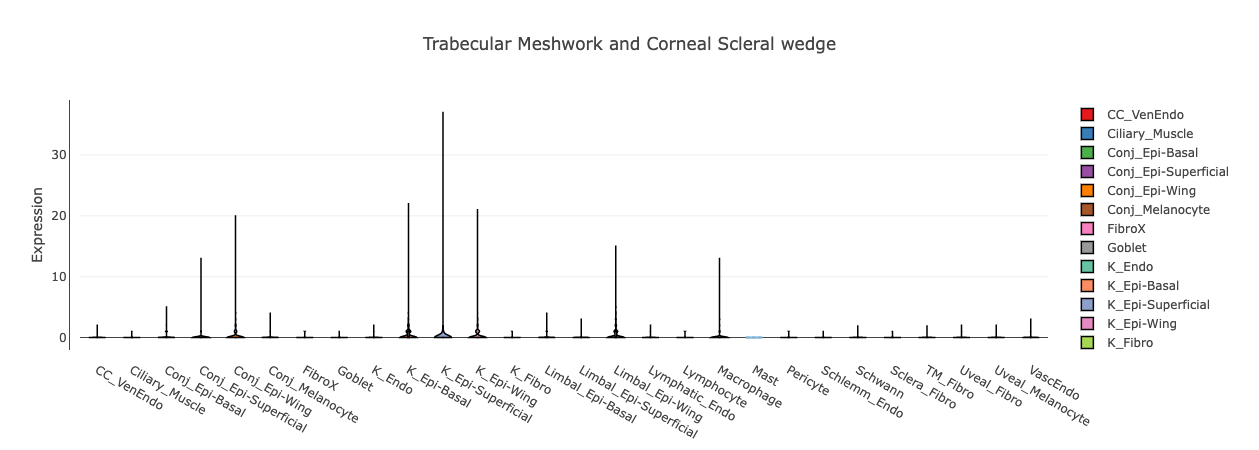
*

*PLK5*

*
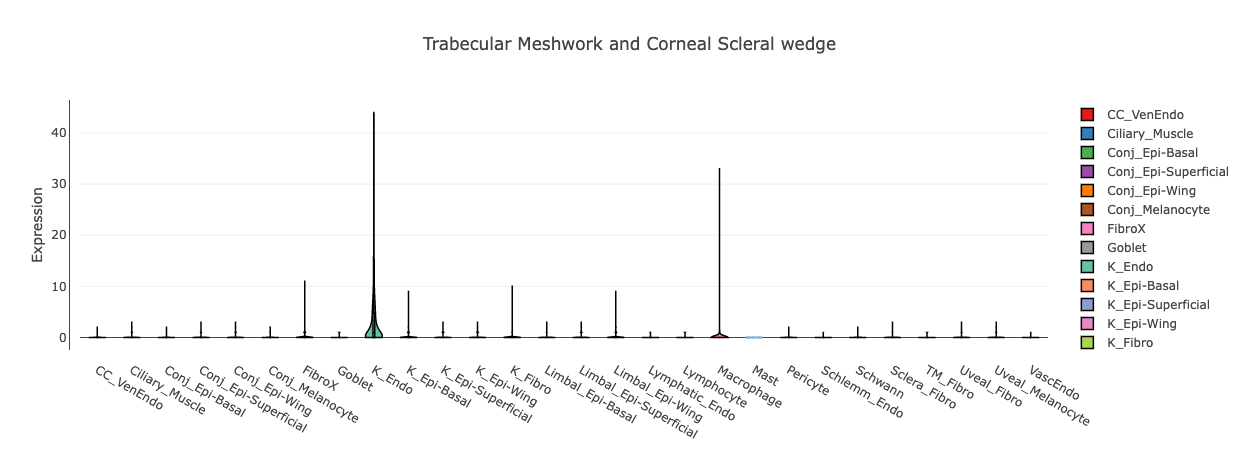
*

*PPIF

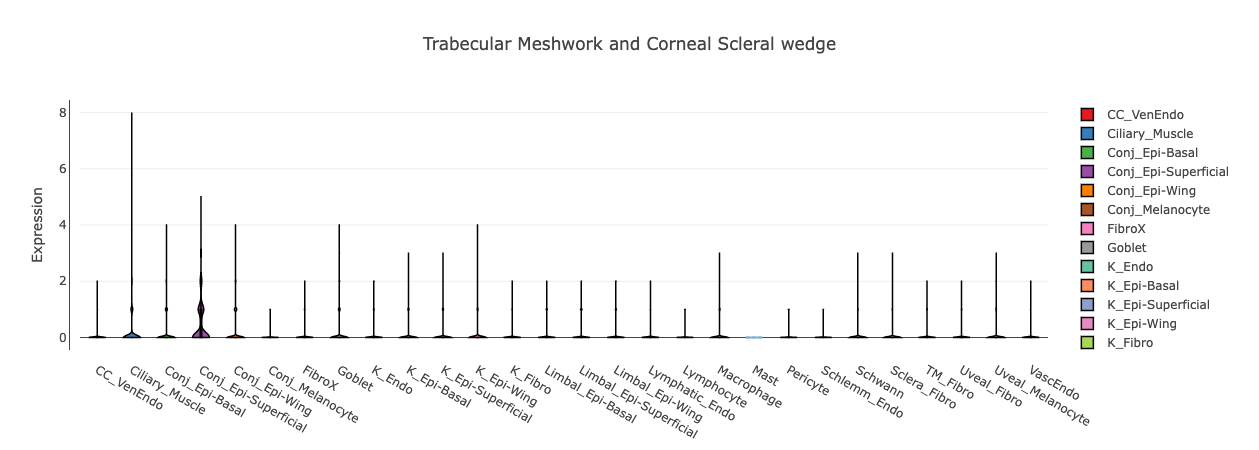
*

*PPM1J*

*
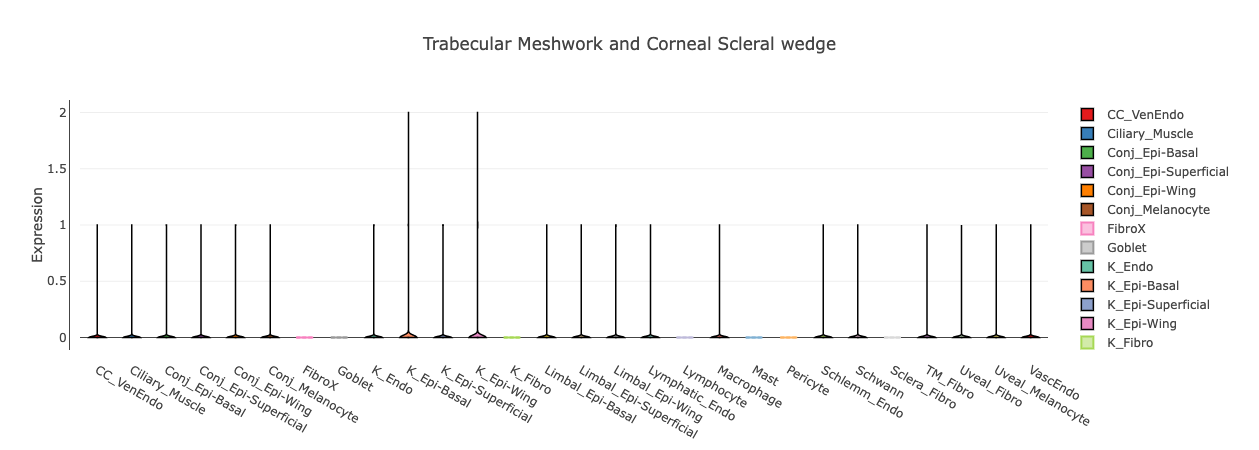
*

*PTPRB*

*
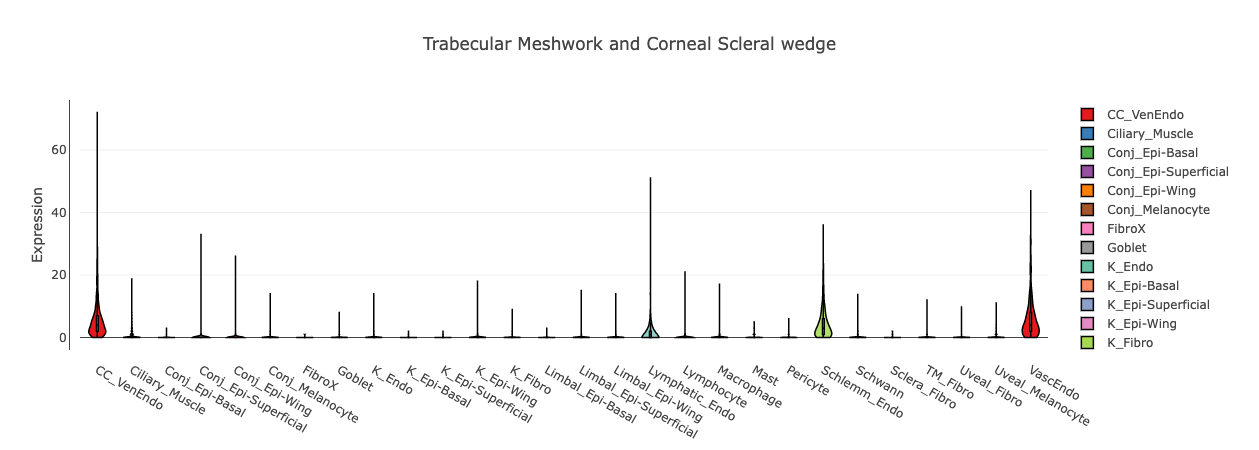
*

*RAB4B-EGLN2*

*
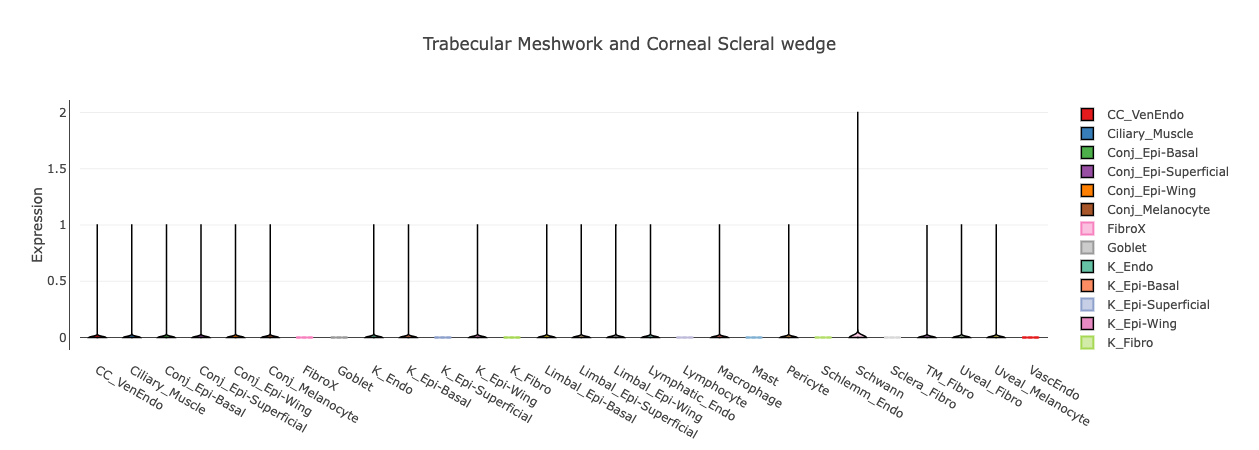
*

*RALYL*

*
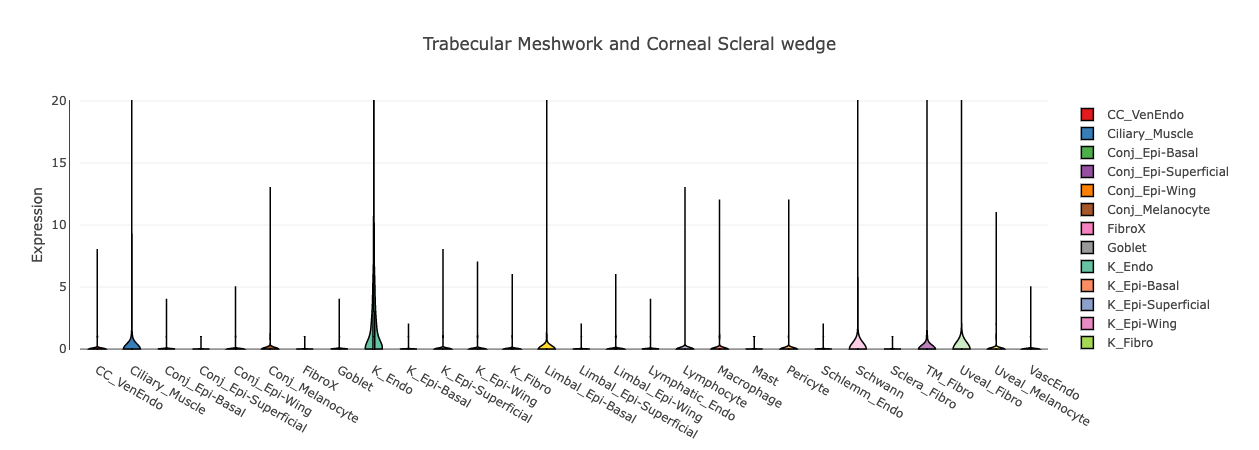
*

*RHOC*

*
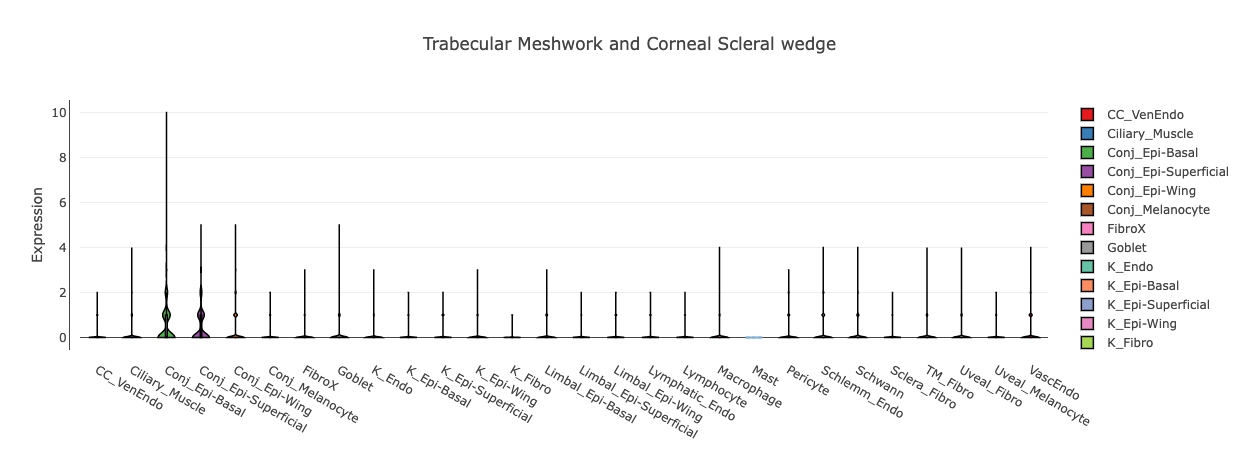
*

*RPL10A*

*
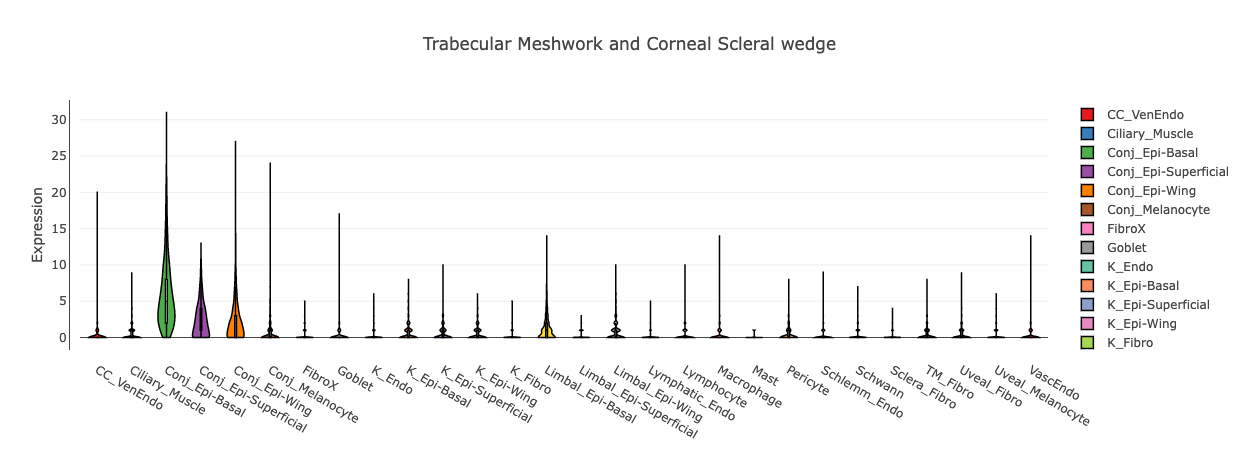
*

*RPL26*

*
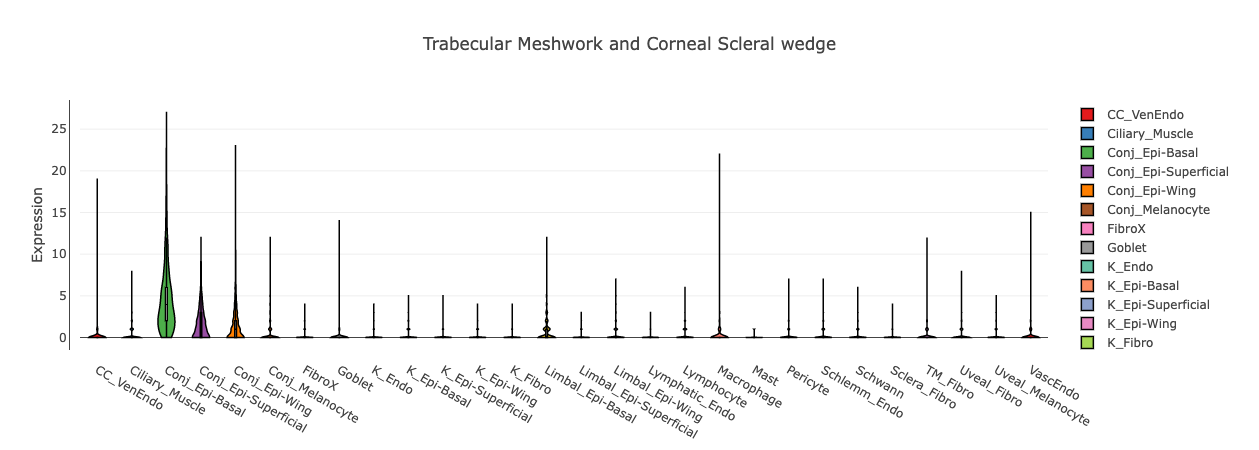
*

*SMG7*

*
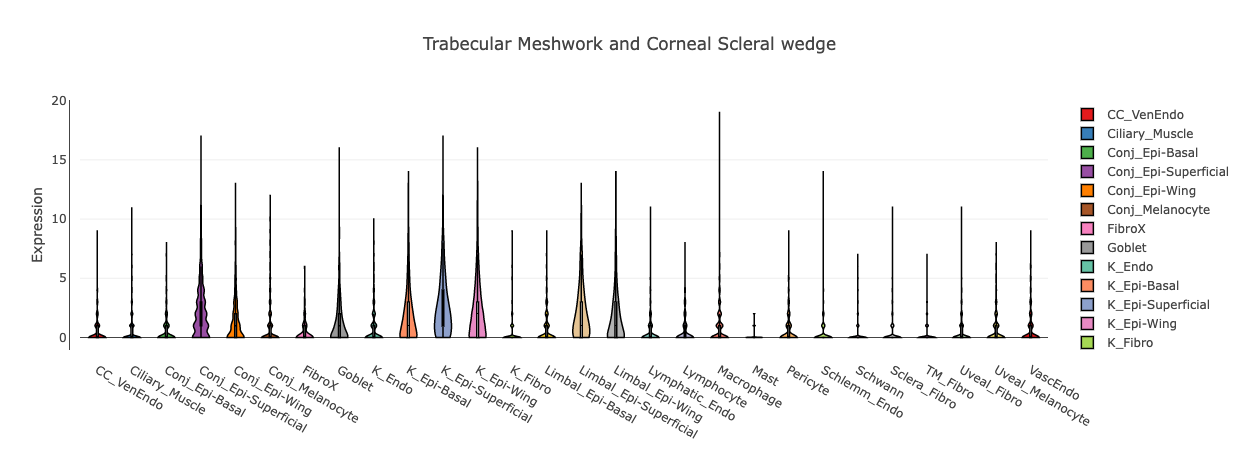
*

*SOS2*

*
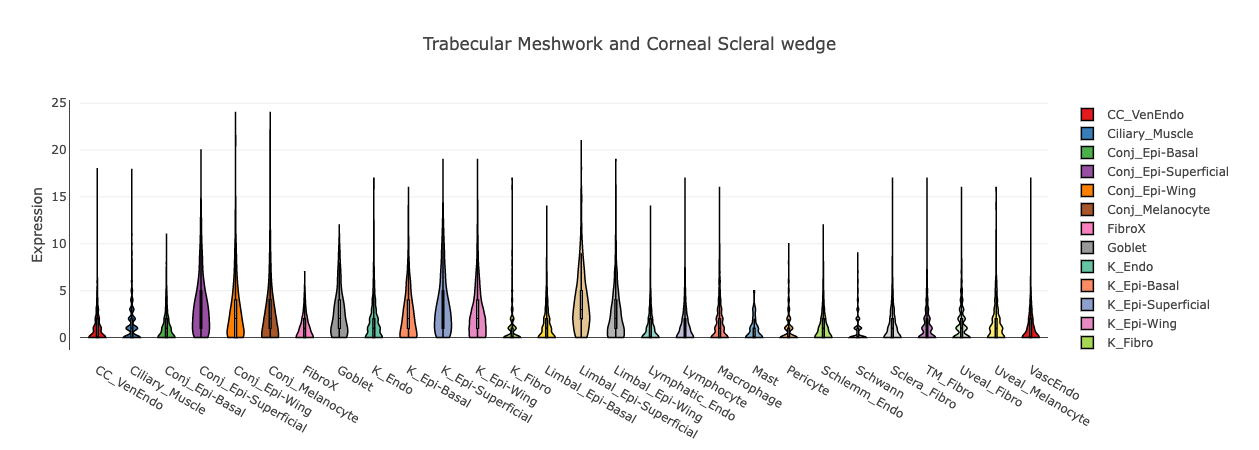
*

*SYNGR3*

*
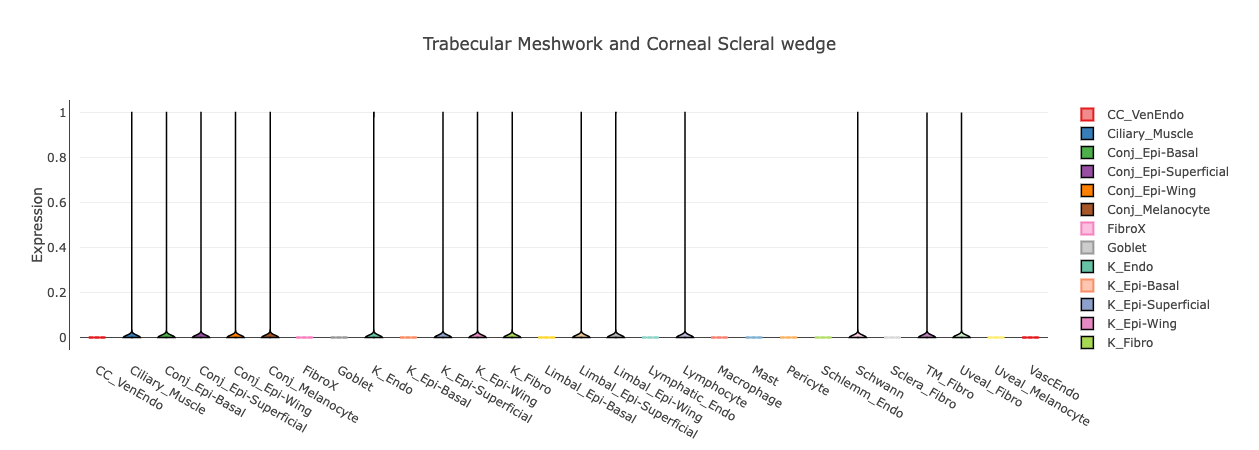
*

*TAF1B*

*
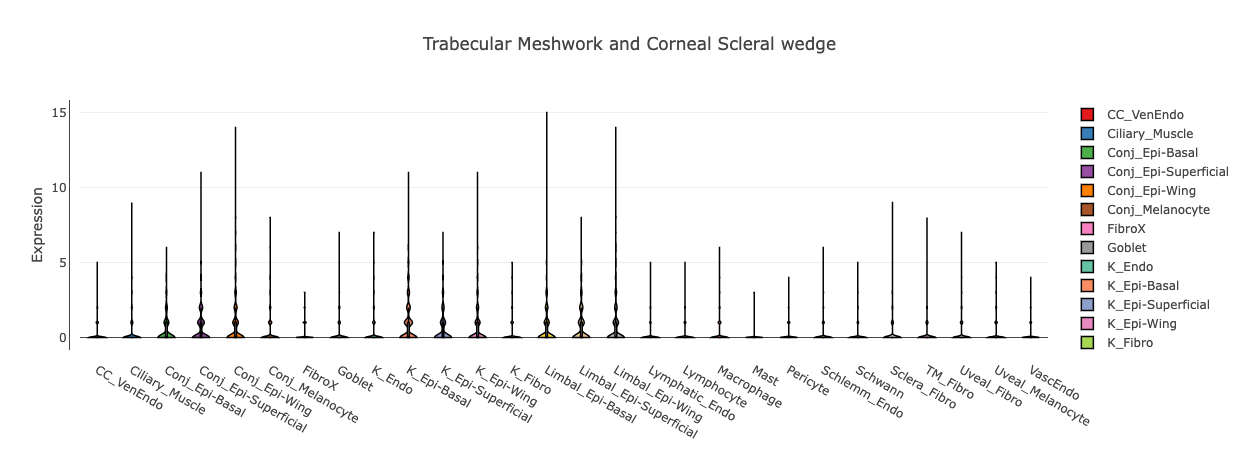
*

*TEAD3*

*
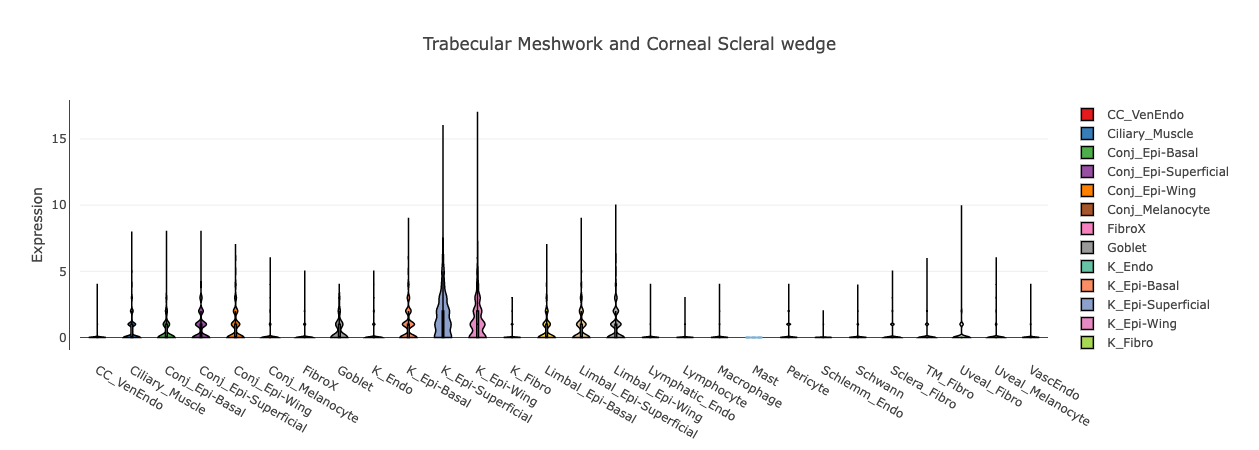
*

*UBN1*

*
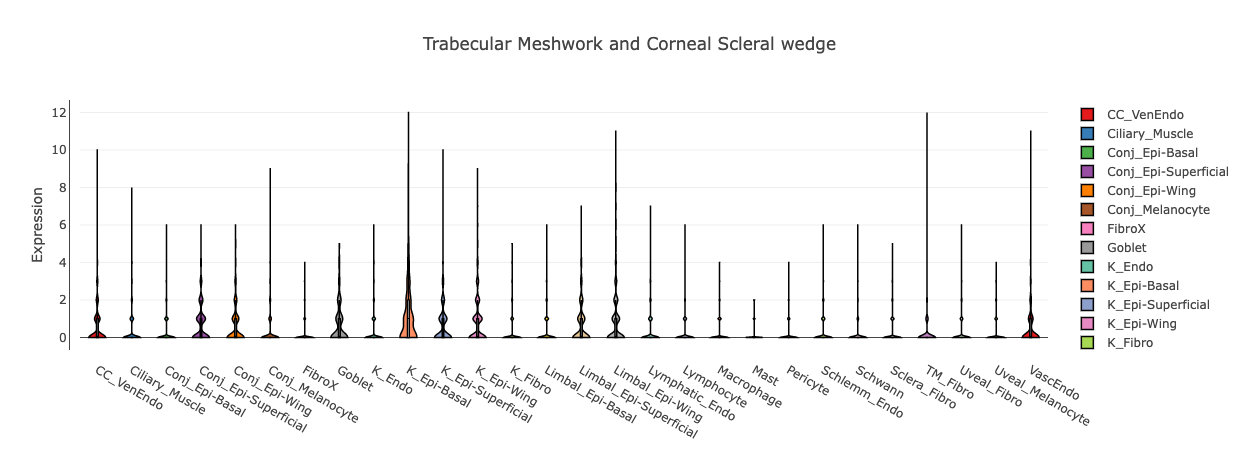
*

*VKORC1L1*

*
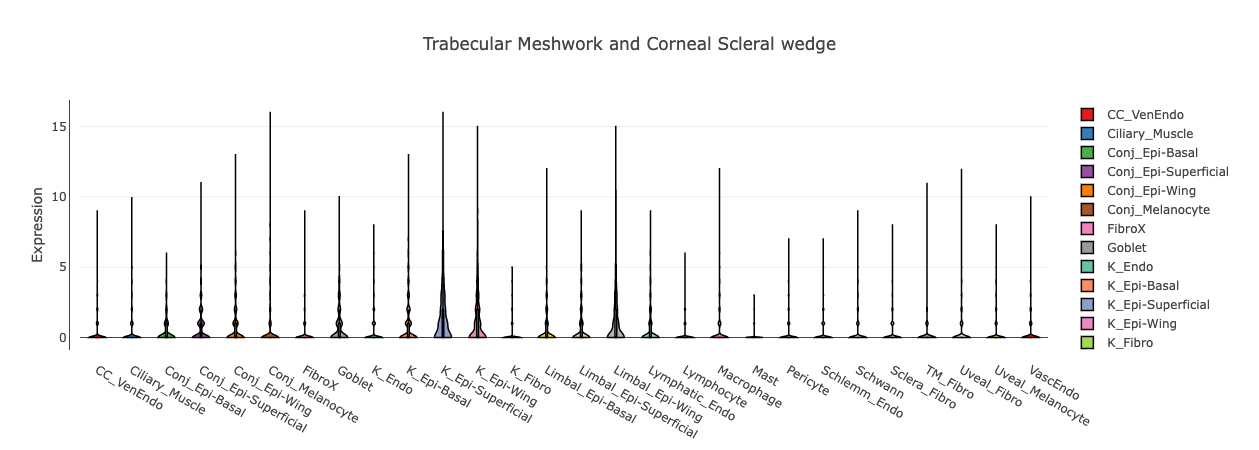
*

*ZNF598*

*
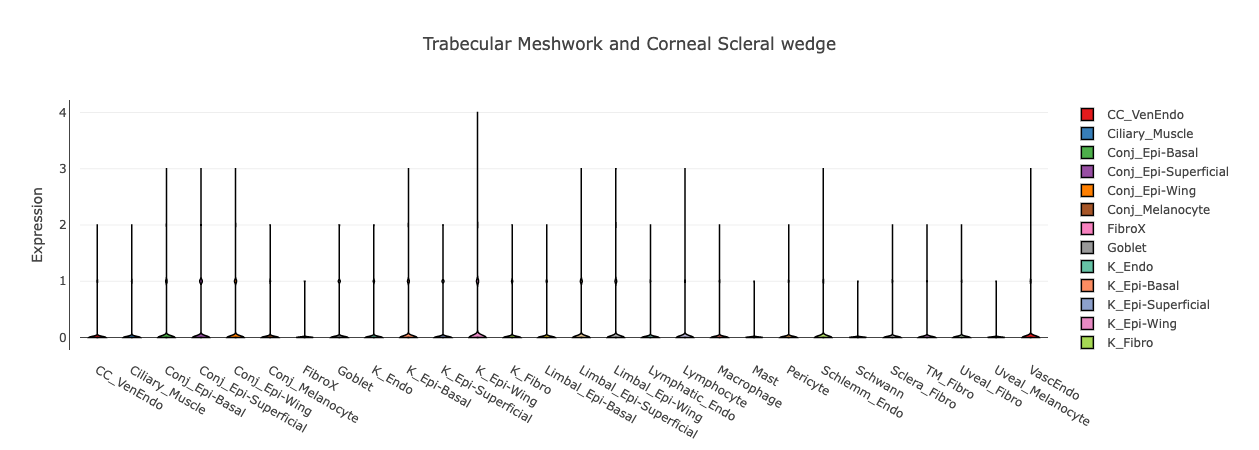
*

**Supplementary Figure 4. Single-cell RNA sequencing profiles from Spectacle.**

We used the Cell atlas of aqueous humor outflow pathways^3^ (queried through Spectacle^4^). Single cell RNA sequencing profiles are listed alphabetically according to gene name.

Abbreviations: Bcell, B cell; BeamCella, Beam Cell A; BeamCellb, Beam Cell B; CiliaryMuscle, Ciliary Muscle; CollectorChnlAqVein, Collector channel aqueous vein; CornealEpi, Corneal epithelium; CribiformJCT, Cribriform juxtacanalicular tissue; Fibroblast, Fibroblast; Macrophage, Macrophage; MastCell, Mast cell; Melanocyte, Melanocyte; Neuron, Neuron; NKT, Natural Killer/T-cell; Pericyte, Pericyte; ScEndo, Schlemm canal endothelium; SchwalbeLine, Schwalbe line; SchwannCell-my, Schwann cell myelinating; SchwannCell-nmy. Schwann cell non-myelinating; VascularEndo, Vascular endothelium.

*AAK1*

*
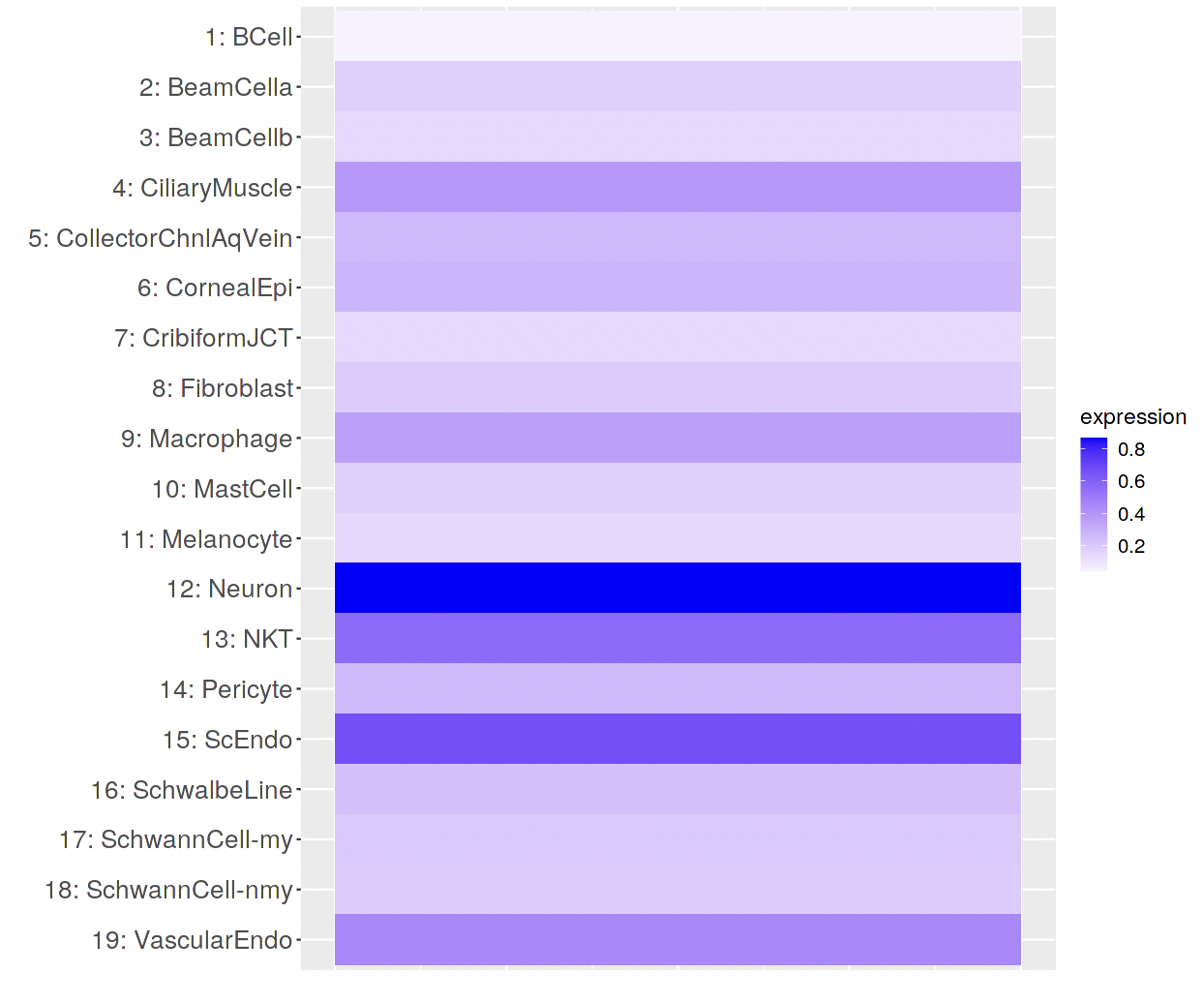
*

*ABI3BP*

*
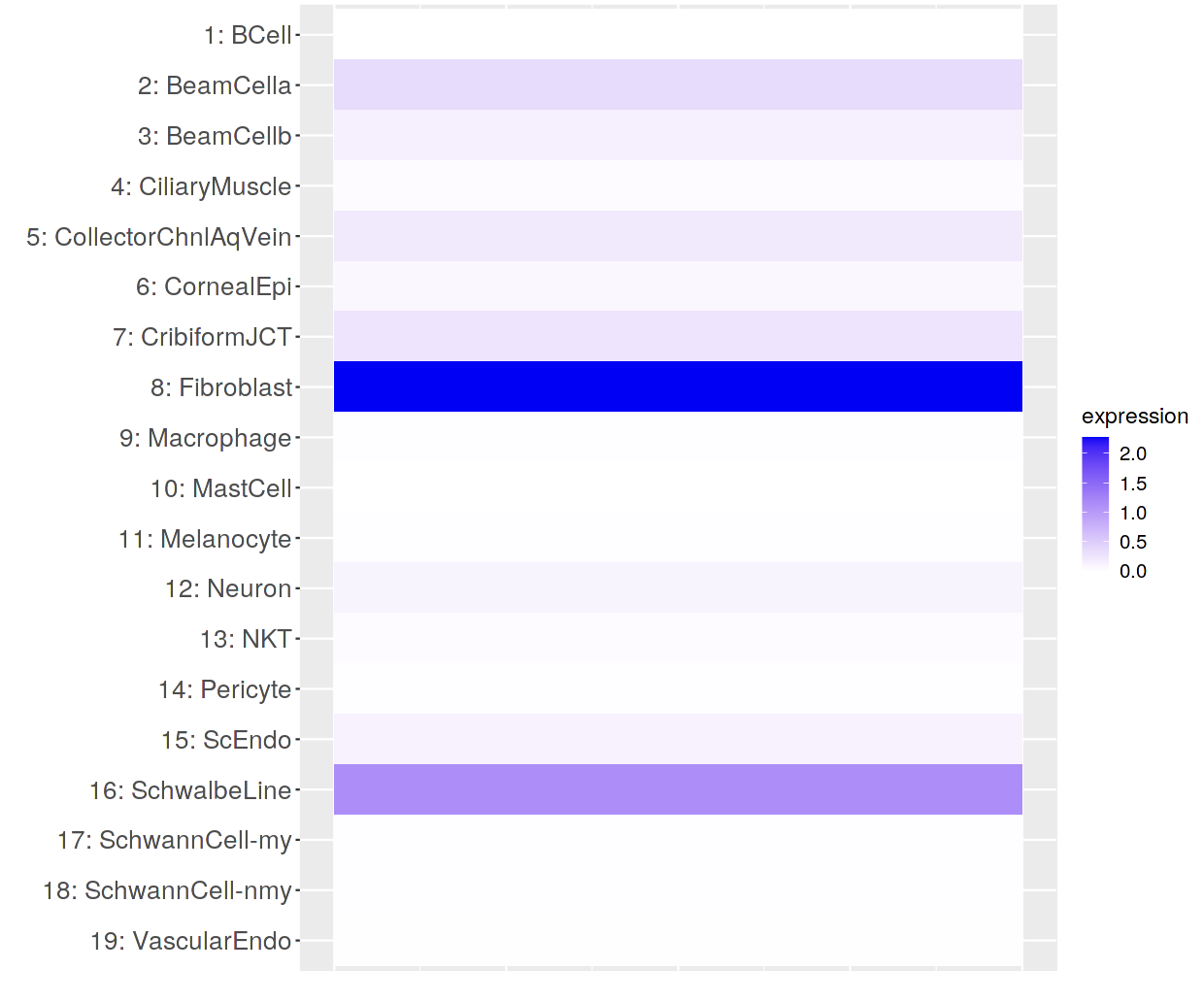
*

*ACAD10*

*
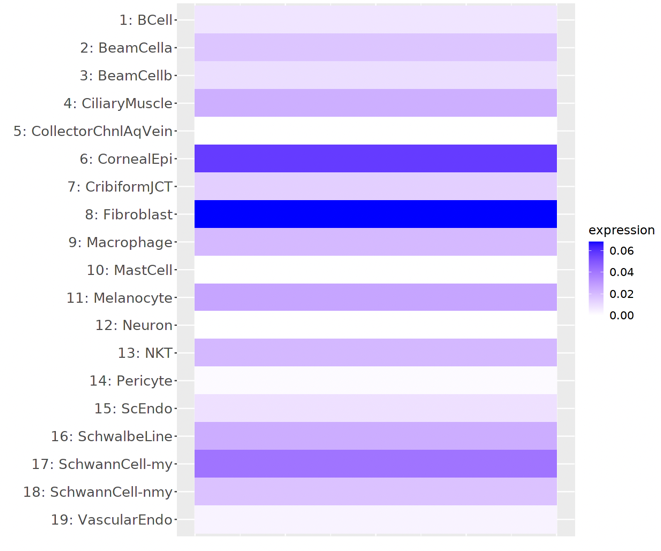
*

*ADRB1*

*ADSS*

*ANGPTL7*

*BOD1L1*

*CDCA8*

*CDK11A*

*DACT3*

*DOK4*

*DPF3*

*EGLN2*

*EHMT1*

*EVA1C*

*FOXD1*

*GUSB*

*HFM1*

*HLA-B*

*IFI27*

*ISY1*

*KIF21A*

*LDB3*

*MTOR*

*MYOC*

*NFXL1*

*PLAU*

*PLK5*

*PPIF*

*PPM1J*

*PTPRB*

*RAB4B*

*RAB43*

*RALYL*

*RHOC*

*RPL10A*

*RPL26*

*SMG7*

*SOS2*

*SYNGR3*

*TAF1B*

*TEAD3*

*UBN1*

*VKORC1L1*

*ZNF598*

Supplementary References

1. Hruz T*, et al.* Genevestigator v3: a reference expression database for the meta-analysis of transcriptomes. *Adv Bioinformatics* **2008**, 420747 (2008).

2. van Zyl T, Yan W, McAdams AM, Monavarfeshani A, Hageman GS, Sanes JR. Cell atlas of the human ocular anterior segment: Tissue-specific and shared cell types. *Proc Natl Acad Sci U S A* **119**, e2200914119 (2022). https://doi.org/10.1073/pnas.2200914119

3. van Zyl T*, et al.* Cell atlas of aqueous humor outflow pathways in eyes of humans and four model species provides insight into glaucoma pathogenesis. *Proc Natl Acad Sci U S A* **117**, 10339-10349 (2020). https://doi.org/10.1073/pnas.2001250117

4. Voigt AP*, et al.* Spectacle: An interactive resource for ocular single-cell RNA sequencing data analysis. *Exp Eye Res* **200**, 108204 (2020). https://doi.org/10.1016/j.exer.2020.108204
