# Supplementary material for: Whole-exome sequencing study identifies rare variants and genes associated with intraocular pressure and glaucoma
Source: Nat Commun. 2022 Nov 30;13:7376. doi: 10.1038/s41467-022-35188-3 (PMC9712679; doi:10.1038/s41467-022-35188-3)
Supplement: Supplementary file 3 — Description of Additional Supplementary Files [file 41467_2022_35188_MOESM3_ESM.pdf]

Description of Additional Supplementary Files

File Name: Supplementary Data 1
Description: Results with $P < 1 \times 10^{-5}$ for treatment with eye drops (if available) were extracted from PhenoScanner queries (GWAS results source: http://www.nealelab.is/uk-biobank). Otherwise, any other eye-related results were extracted. About two thirds of the genes in the table are associated with glaucoma topical treatments, while about one half are associated with dry eye treatments. It was reported that glaucoma and dry eye may commonly occur together[1].

[1]Nijm LM, De Benito-Llopis L, Rossi GC, Vajaranant TS, Coroneo MT. Understanding the Dual Dilemma of Dry Eye and Glaucoma: An International Review. *Asia Pac J Ophthalmol (Phila)* **9**, 481-490 (2020).

File Name: Supplementary Data 2
Description: The results from phenome-wide association study on the genes found in rare-variant and gene-based analyses are shown. From PheWeb top hits list, we extracted eye related, cardiovascular, and nervous system related phenotypes if available, otherwise the top hits were extracted.

File Name: Supplementary Data 3
Description: Abbreviations: Chr, chromosome; REF, reference allele; ALT, alternative allele; ALT_FREQS, alternative allele frequencies; ALT_CTS, alternative allele counts; OBS_CT, observation count.
