# Supplementary material for: Whole-exome sequencing study identifies rare variants and genes associated with intraocular pressure and glaucoma
Source: Nat Commun. 2022 Nov 30;13:7376. doi: 10.1038/s41467-022-35188-3 (PMC9712679; doi:10.1038/s41467-022-35188-3)
Supplement: Supplementary file 7 — Reporting Summary [file 41467_2022_35188_MOESM7_ESM.pdf]

Corresponding author(s): Xiaoyi Raymond Gao
Last updated by author(s): Oct 21, 2022

Reporting Summary

Nature Portfolio wishes to improve the reproducibility of the work that we publish. This form provides structure for consistency and transparency in reporting. For further information on Nature Portfolio policies, see our Editorial Policies and the Editorial Policy Checklist.

Statistics

For all statistical analyses, confirm that the following items are present in the figure legend, table legend, main text, or Methods section.

n/a	Confirmed	
☐	☒	The exact sample size (n) for each experimental group/condition, given as a discrete number and unit of measurement
☐	☒	A statement on whether measurements were taken from distinct samples or whether the same sample was measured repeatedly
☐	☒	The statistical test(s) used AND whether they are one- or two-sided<br>*Only common tests should be described solely by name; describe more complex techniques in the Methods section.*
☐	☒	A description of all covariates tested
☐	☒	A description of any assumptions or corrections, such as tests of normality and adjustment for multiple comparisons
☐	☒	A full description of the statistical parameters including central tendency (e.g. means) or other basic estimates (e.g. regression coefficient) AND variation (e.g. standard deviation) or associated estimates of uncertainty (e.g. confidence intervals)
☐	☒	For null hypothesis testing, the test statistic (e.g. F, t, r) with confidence intervals, effect sizes, degrees of freedom and P value noted<br>*Give P values as exact values whenever suitable.*
☒	☐	For Bayesian analysis, information on the choice of priors and Markov chain Monte Carlo settings
☒	☐	For hierarchical and complex designs, identification of the appropriate level for tests and full reporting of outcomes
☐	☒	Estimates of effect sizes (e.g. Cohen's d, Pearson's r), indicating how they were calculated

Our web collection on statistics for biologists contains articles on many of the points above.

Software and code

Policy information about availability of computer code

Data collection	No software was used for data collection.
Data analysis	We used the REGENIE v2.2.4 software for single variant analyses, and SAIGE-GENE v0.44.1 for gene-based analyses. We also used ANNOVAR (2020 June 08 version), VEP v100, PLINK v1.9 and R v4.0 for the rest of our analyses. The URLs for the programs used: ANNOVAR, http://annovar.openbioinformatics.org/; REGENIE, https://rgcgithub.github.io/regenie/; SAIGE, https://github.com/weizhouUMICH/SAIGE; VEP, https://useast.ensembl.org/info/docs/tools/vep/index.html; R version 4.0, (https:// www.r-project.org), PLINK, https://www.cog-genomics.org/plink/.

For manuscripts utilizing custom algorithms or software that are central to the research but not yet described in published literature, software must be made available to editors and reviewers. We strongly encourage code deposition in a community repository (e.g. GitHub). See the Nature Portfolio guidelines for submitting code & software for further information.

Data

Policy information about availability of data

All manuscripts must include a data availability statement. This statement should provide the following information, where applicable:

- Accession codes, unique identifiers, or web links for publicly available datasets
- A description of any restrictions on data availability
- For clinical datasets or third party data, please ensure that the statement adheres to our policy

The UK Biobank data, both phenotypic and genetic, used in this study are available in the UK Biobank database and was accessed under application number 23424 (https://www.ukbiobank.ac.uk).
The intraocular pressure summary statistics generated in this study are available at https://github.com/xraygao/GWAS_results.
The following are links to public datasets we used in this study:
ChEMBL, https://www.ebi.ac.uk/chembl/
FinnGen, https://www.finngen.fi/
Genevestigator, https://genevestigator.com/
PhenoScanner, http://www.phenoscanner.medschl.cam.ac.uk/
PheWeb, https://pheweb.sph.umich.edu/
Spectacle, https://singlecell-eye.org/app/spectacle/
The Broad Institute's Single Cell Portal, https://singlecell.broadinstitute.org/single_cell/study/SCP1841/
UK Biobank, https://www.ukbiobank.ac.uk

Human research participants

Policy information about studies involving human research participants and Sex and Gender in Research.

Reporting on sex and gender	Among the IOP whole-exome squencing (WES) data used in our IOP study, 54% of the participants were female. Sex was included as a covariate in our analyses. We did not perform sex-specific analysis in this study.
Population characteristics	The UKB recruited over 500,000 adult participants (40 to 70 years of age at enrollment) living in the United Kingdom who were registered with the National Health Service at the study baseline (from 2006 to 2010). Medical information (self-report and electronic health records), family history, lifestyle information, as well as DNA samples, were collected. Ophthalmological data were also collected for a subset of study participants (~118,000). Most participants (~94%) reported their ethnic background as white and the rest originated outside of Europe. A total of 110,260 UKB participants were included in the IOP WES analysis, of which 98,674 were white. The mean (standard deviation [SD]) of age was 58 (8.1) years and 54% of the participants were female.
Recruitment	Participants were recruited by UKB as described here: https://www.ukbiobank.ac.uk/enable-your-research/about-our-data/baseline-assessment.
Ethics oversight	UKB was approved by the North West Multi-Center Research Ethics Committee. Our access of UKB data was approved under application #23424.

Note that full information on the approval of the study protocol must also be provided in the manuscript.

Field-specific reporting

Please select the one below that is the best fit for your research. If you are not sure, read the appropriate sections before making your selection.

☒ Life sciences ☐ Behavioural & social sciences ☐ Ecological, evolutionary & environmental sciences

For a reference copy of the document with all sections, see nature.com/documents/nr-reporting-summary-flat.pdf

Life sciences study design

All studies must disclose on these points even when the disclosure is negative.

Sample size	We used all available data in UKB after quality control. Sample size was determined using the data exclusions below. Any sample not excluded based on those criteria were considered samples. Overlapping with WES data, a total of 110,260 UKB participants were included in the IOP WES analysis, of which 98,674 were white.
Data exclusions	The average of both eyes was used for downstream analysis. If only one IOP measurement was obtained, it was used as the final value. Study participants who received eye surgery within 4 weeks prior to the ocular assessment or those with possible eye infections did not receive IOP measurements. Moreover, we excluded study participants with extreme values of IOP, i.e., in the bottom and top 0.3 percentiles, and outliers, including participants who had either eye surgery or used eye drop medications.
Replication	Replication was done using the publicly available summary statistics results FinnGen, PhenoScanner, and PheWeb (links above). P-values for

Replication	the queried genes were provided. We carried out replications once for all our exome-wide significant hits. We found replications of our queried genes in FinnGen, PhenoScanner, and PheWeb. Not all genes were found in these databases or if found their p-values may not have been significant enough to be considered successful replication. Thus not all attempts at replication were successful.
Randomization	Given the exposure in our analysis is genetic variants and the phenotypes were collected independently, individuals were not experimentally randomized.
Blinding	We were blinded to group allocation during data analysis as we used de-identified samples only. We did not perform data collection, as that was carried out by UKB. We are not aware any specific blinding was used in UKB human sample collection.

Reporting for specific materials, systems and methods

We require information from authors about some types of materials, experimental systems and methods used in many studies. Here, indicate whether each material, system or method listed is relevant to your study. If you are not sure if a list item applies to your research, read the appropriate section before selecting a response.

Materials & experimental systems

n/a	Involved in the study
☒	☐ Antibodies
☒	☐ Eukaryotic cell lines
☒	☐ Palaeontology and archaeology
☒	☐ Animals and other organisms
☒	☐ Clinical data
☒	☐ Dual use research of concern

Methods

n/a	Involved in the study
☒	☐ ChIP-seq
☒	☐ Flow cytometry
☒	☐ MRI-based neuroimaging
